# A machine learning-derived gene signature for assessing rupture risk and circulatory immunopathologic landscape in patients with intracranial aneurysms

**DOI:** 10.3389/fcvm.2023.1075584

**Published:** 2023-02-10

**Authors:** Taoyuan Lu, Yanyan He, Zaoqu Liu, Chi Ma, Song Chen, Rufeng Jia, Lin Duan, Chunguang Guo, Yiying Liu, Dehua Guo, Tianxiao Li, Yingkun He

**Affiliations:** ^1^Department of Cerebrovascular Disease and Neurosurgery, Zhengzhou University People’s Hospital, Henan Provincial People’s Hospital, Zhengzhou, Henan, China; ^2^Henan International Joint Laboratory of Cerebrovascular Disease, Henan Provincial NeuroInterventional Engineering Research Center, Henan Engineering Research Center of Cerebrovascular Intervention Innovation, Zhengzhou, China; ^3^Department of Interventional Radiology, The First Affiliated Hospital of Zhengzhou University, Zhengzhou, Henan, China; ^4^Translational Research Institute, Henan Provincial People’s Hospital, Zhengzhou, Henan, China; ^5^Department of Endovascular Surgery, The First Affiliated Hospital of Zhengzhou University, Zhengzhou, Henan, China; ^6^Department of Rehabilitation Medicine, The First Affiliated Hospital of Zhengzhou University, Zhengzhou, Henan, China

**Keywords:** intracranial aneurysm, machine learning, multigene model, rupture risk, immunopathological features, precision medicine

## Abstract

**Background:**

Intracranial aneurysm (IA) is an uncommon but severe subtype of cerebrovascular disease, with high mortality after aneurysm rupture. Current risk assessments are mainly based on clinical and imaging data. This study aimed to develop a molecular assay tool for optimizing the IA risk monitoring system.

**Methods:**

Peripheral blood gene expression datasets obtained from the Gene Expression Omnibus were integrated into a discovery cohort. Weighted gene co-expression network analysis (WGCNA) and machine learning integrative approaches were utilized to construct a risk signature. QRT-PCR assay was performed to validate the model in an in-house cohort. Immunopathological features were estimated using bioinformatics methods.

**Results:**

A four-gene machine learning-derived gene signature (MLDGS) was constructed for identifying patients with IA rupture. The AUC of MLDGS was 1.00 and 0.88 in discovery and validation cohorts, respectively. Calibration curve and decision curve analysis also confirmed the good performance of the MLDGS model. MLDGS was remarkably correlated with the circulating immunopathologic landscape. Higher MLDGS scores may represent higher abundance of innate immune cells, lower abundance of adaptive immune cells, and worse vascular stability.

**Conclusions:**

The MLDGS provides a promising molecular assay panel for identifying patients with adverse immunopathological features and high risk of aneurysm rupture, contributing to advances in IA precision medicine.

## Introduction

Intracranial aneurysms (IAs) are local saccular dilatations within cerebrovascular driven by complex pathological factors, with low prevalence but high mortality and disability rate (1). The high mortality and disability rate can be mainly attributed to intrinsic adverse molecular pathological changes and aneurysm rupture (2). Preventive endovascular or neurosurgical treatment can pronouncedly reduce the rate of aneurysm rupture and improve overall survival (3–5); however, these treatments also carry a substantial risk of procedural complications (6) and a heavy economic burden. In some patients with “low-risk” IAs, the aneurysms are only dynamically observed without being treated as the risk of complications is evaluated to be higher than the risk of rupture. Hence, recognizing patients with “high-risk” IAs is critical for clinical management decisions. In the current clinical setting, the risk and treatment demands of a specific patient are usually assessed by clinical and imaging characteristics, such as age, hypertension, morphological parameters, and specific magnetic resonance angiography features (7–10). Yet, these approaches are limited by moderate accuracy, radiation exposure, and disregard of molecular pathological heterogeneity, which is intimately related to the risk of rupture and the onset of relevant complications such as cerebral vasospasm (CVS) (11, 12). In the era of precision medicine, it is imperative to identify available molecular biomarkers for optimizing individualized risk assessment system in patients with IAs.

Intracranial aneurysms is a complex disease with incompletely ascertained molecular pathophysiology. According to the current knowledge, immune inflammation, extracellular matrix (ECM) metabolism, and vascular smooth muscle cell loss have essential roles in vessel wall disruption, which is closely associated with the progression and rupture of IAs (1, 13). In addition, dysregulation of vasomotor regulation-related molecules, such as endothelial nitric oxide synthase (eNOS) and endothelin-1, is an important pathogenic mechanism for CVS after aneurysmal subarachnoid hemorrhage (14–16). Mastery of these molecular pathological data will facilitate more precise individualized risk assessment in patients with IAs. In recent years, progress in high-throughput and bioinformatics technologies has provided novel opportunities for decoding the detailed molecular alterations of IAs, thereby identifying a range of potential causative genes or biomarkers (17–19). However, tissue-based biomarkers are usually difficult to apply in clinical settings because of the limited sampling. Considering the convenience of sampling and the multimolecular driving properties of the disease, a blood-based multigene panel might be an ideal molecular detection technique for IA auxiliary diagnosis or risk evaluation.

Taken together with the aforementioned considerations, the present study attempted to develop and validate a novel machine learning-derived gene signature (MLDGS) for assessing aneurysm rupture risk and circulatory immunopathological landscape in patients with IAs. Combined machine learning approaches were applied to fit models in an integrated public dataset, and an in-house clinical cohort was recruited for model validation. Our findings may contribute to optimizing the precise diagnosis and treatment system, and further improving the clinical outcomes of IA patients.

## Materials and methods

### Publicly available datasets collection and preprocessing

**FIGURE 1:**
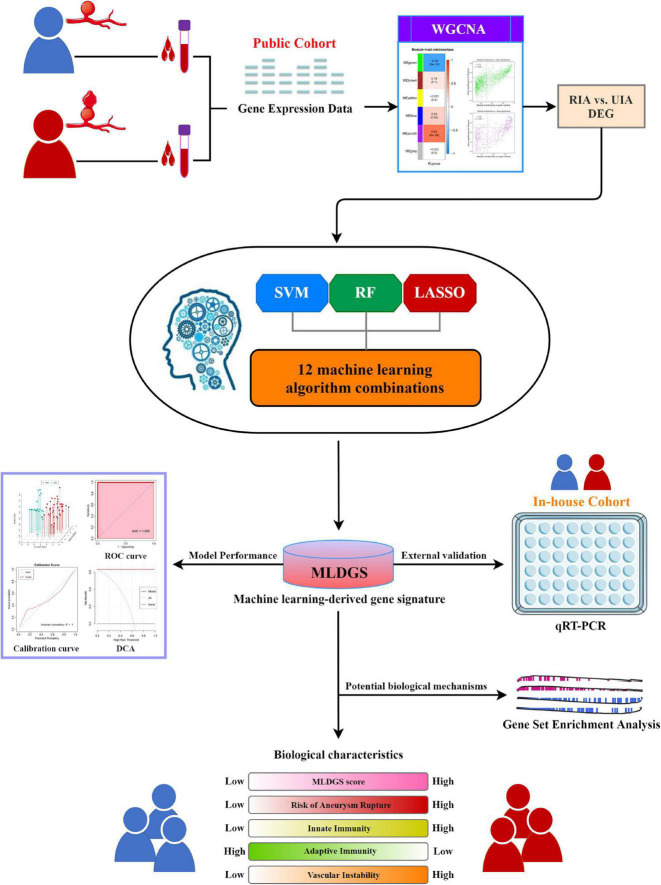
The overall workflow of this study.

The overall workflow of this study is illustrated in Figure 1. Peripheral blood gene expression datasets GSE36791 (43 RIA patients and 18 IA-free subjects) and GSE159610 (25 UIA patients and 22 IA-free subjects) were collected from the Gene Expression Omnibus (GEO)^1^ database. The detailed baseline information is listed in Supplementary Table 1. The RNA-seq raw read counts in GSE159610 were converted to transcripts per kilobase million (TPM) values and further log-2 transformed, which is more similar to those resulting from microarrays and more comparable between samples. The GENCODE (Homo sapiens GRCh38) was utilized for RNA annotations. The microarray dataset GSE36791 based on Illumina ^®^ platforms (GPL10558) was quantile normalized and further log2 transformed via the *limma* R package. Subsequently, GSE36791 and GSE159610 were integrated into one cohort (denoted as the public cohort) after removing batch effects via the *ComBat* algorithm implemented in the *sva* R package (20). Only patients with RIAs or UIAs were included in this study.

### Weighted gene co-expression network analysis (WGCNA)

Weighted gene co-expression network analysis (WGCNA), an advanced bioinformatics method capable of identifying gene interrelationships and gene-phenotype relationships, was utilized to recognize gene co-expression modules intimately related to IA rupture. Similar to previous studies (21, 22), top 5,000 genes selected according to the median absolute deviation were employed to construct co-expression networks based on weighted correlation adjacency matrix and cluster analysis via the *WGCNA* R package (23). An appropriate soft threshold β was calculated via the “pickSoftThreshold” function to meet the criteria for scale-free network and construct a weighted adjacency matrix. Next, the weighted adjacency matrix was converted into a topological overlap matrix (TOM), and the corresponding dissimilarity (1-TOM) was generated. The dynamic tree cutting approach (corType = “pearson”, minModuleSize = 50) was utilized for module recognition, and modules with less than 0.25 dissimilarity were merged. Modules that displayed the highest correlation with IA rupture were extracted for further investigation. To identify hub genes within the modules, genes with both high gene significance (>0.5) and module membership (>0.5) were extracted as hub rupture-associated genes (RAGs).

### Gene differential expression analysis

The *limma* R package was utilized to identify differentially expressed genes (DEGs) between RIAs and UIAs, with the absolute value of log2 fold change (logFC) > 1 and false discovery rate (FDR) < 0.05 as the screening criteria. DEGs overlapping with the RAGs were selected as input feature gene variables for subsequent machine learning modeling.

### Machine learning-based integrative approaches for signature generation

Prior to constructing the MLDGS model, gene relative expression values were transformed into z-scores in the public cohort, thus enhancing the comparability between different datasets. To develop an accurate machine learning-derived model for recognizing RIAs, we integrated three classic machine learning algorithms and 12 algorithm combinations, similar to Liu et al. (22, 24). The three basic algorithms included random forest (RF), support vector machine (SVM), and least absolute shrinkage and selection operator (LASSO) regression. Considering these algorithms possessed the ability of dimension reduction and feature selection, we combined these algorithms to generate several integrative models, including RF + SVM, RF + LASSO, SVM + RF, SVM + LASSO, LASSO + SVM, LASSO + RF, RF + SVM + LASSO, SVM + LASSO + RF, and RF + LASSO + SVM. Briefly, in each combination, the feature variables were pre-screened using the top-ranked algorithms, after which the model was constructed using the last algorithm. The detailed signature development procedure was as follows: (a) The public cohort was randomly divided into training set and validation set at a 7:3 ratio. (b) 12 algorithm combinations were performed on the input feature gene variables to fit dichotomous models based on the leave-one-out cross-validation (LOOCV) framework in the training set. (c) All models were also detected in the validation set and all set. (d) For each model, the number of model genes, and the accuracy, sensitivity, specificity, precision, and negative predictive value (NPV) for distinguishing RIAs were calculated in the training set, validation set and all set, respectively. The model with the highest average accuracy and the least number of model genes was considered optimal. Furthermore, receiver operating characteristic (ROC) curve, calibration curve and decision curve analysis (DCA) were employed to evaluate the performance of the optimal model. The SVM, RF, and LASSO regression were implemented via the *e1071*, *randomForest*, and *glmnet* R packages, respectively. The ROC curve, calibration curve, and DCA were implemented via the *pROC*, *rms*, and *rmda* R packages, respectively.

### Blood sample collection

This study was approved by the Ethical Committee of Zhengzhou University People’s Hospital, China. All patients were aged >18 years and gave written informed consent. A total of 28 peripheral blood samples (RIAs vs. UIAs = 13:15, denoted as in-house cohort) were collected from the Department of Cerebrovascular Disease, Zhengzhou University People’s Hospital. Blood samples were collected and temporarily stored in the EDTA anticoagulant tubes prior to subsequent RNA extraction. All patients with UIA or RIA were diagnosed by digital subtraction angiography and were not previously treated for IA. Patients who had a fever, tumor or autoimmune diseases, had recently invasive surgery, or were receiving chemotherapy or immunomodulating drugs, as noted in their medical records, were excluded. The detailed baseline information is listed in Supplementary Table 1.

### Quantitative real-time polymerase chain reaction (qRT-PCR)

Quantitative real-time polymerase chain reaction (qRT-PCR) was applied to detect the expression of model genes in peripheral blood samples from our in-house cohort. Total RNA was isolated from peripheral blood samples using RNAprep Pure Hi-Blood Kit (TIANGEN, China) according to the manufacturer’s instructions. An aliquot of 1 μg of total RNA was reverse-transcribed into complementary DNA (cDNA) using HiScript^®^ III RT SuperMix for qPCR (Vazyme, China) according to the manufacturer’s protocol. ChamQ Universal SYBR qPCR Master Mix (Vazyme, China) was applied in qRT-PCR experiments. The expression level was quantized by 2^–ΔΔCt^ mode. GAPDH serves as an internal reference for normalization. The primer sequences for qRT-PCR are illustrated in Supplementary Table 2.

### Estimation of immune cell infiltration

According to the expression level of immune cell-specific marker genes (25), the relative abundance of 28 immune cells in each sample was quantified by single sample gene set enrichment analysis (ssGSEA) algorithm implemented in the *GSVA* R package (26), which is broadly utilized in immune infiltration-relevant bioinformatics studies (27–30). The detail of the gene sets marking 28 immune cells was listed in the Supplementary Table 3.

### Collection of immune-related gene sets and genes regarding vascular stability and brain injury

To investigate the expression patterns of immune-related genes in the peripheral blood of IA patients, we searched the ImmPort database^2^ and collected 17 immune-related gene sets (Additional file: Supplementary Table 4) encompassing antigen processing and presentation, BCR signaling pathway, TCR signaling pathway, NK cell cytotoxicity, chemokines, cytokines, interferons, interleukins, TGF-b family members, TNF family members, and corresponding receptors. Moreover, some vascular stability relevant genes including *IL1B*, *TLR4*, *VEGFA*, *MMP9*, *TIMP1*, *NOS3*, *EDN1*, *ANGPT1* and *ANGPT2* (1, 31–36), as well as brain injury relevant biomarkers such as *UCHL1*, *S100B* and *MBP* (37), were recruited from previous studies to explore the ability of the MLDGS to assess vascular stability and brain injury. Among these genes, *IL1B*, *TLR4*, *VEGFA*, *MMP9*, and *TIMP1* were associated with vascular inflammation and extracellular matrix metabolism; *ANGPT1* and *ANGPT2*, encoding angiopoietin (Ang) I and II, were associated with vascular homeostasis; *NOS3* and *EDN1*, encoding eNOS and endothelin-1, respectively, were associated with vasomotor function and CVS; and *UCHL1*, *S100B* and *MBP* were reported to be prognostic biomarkers for brain injury.

### Gene set variation analysis (GSVA)

Gene set variation analysis (GSVA) was performed according to the immune-related gene sets via the *GSVA* R package. Each sample obtained a set of enrichment scores corresponding to these gene sets based on the gene expression profile. And then, the *limma* R package was employed to compare GSVA enrichment scores between RIAs and UIAs, identifying dysregulated immune-related gene families.

### Gene set enrichment analysis (GSEA) for the MLDGS

A total of 7,667 gene sets were obtained from the MSigDB resource (version 7.4, c2.cp.kegg.v7.4.entrez.gmt and c5.go.bp.v7.4.entrez.gmt). The correlation coefficients between the MLDGS score and all mRNAs were calculated. Subsequently, all mRNAs were sorted in descending order based on their correlations with the MLDGS score. The ranked gene list was further subjected to the *clusterProfiler* R package to perform GSEA. Permutations were set to 10,000 to obtain normalized enrichment scores in GSEA. Gene sets with an FDR < 0.01 were considered to be significantly enriched.

### Statistical analysis

All data processing, statistical analysis, and plotting were conducted in R 4.1.0 software. Significance was assessed via Student’s *t*-test or Wilcoxon rank-sum test for comparisons of two groups and Kruskal–Wallis test for comparisons of three or more groups. Correlations between two continuous variables were determined using Pearson correlations. All heatmaps were created via the *ComplexHeatmap* R package. All statistical tests were two-tailed and *P* < 0.05 was considered statistically significant.

## Results

### Identification of rupture-associated gene modules by WGCNA

**FIGURE 2:**
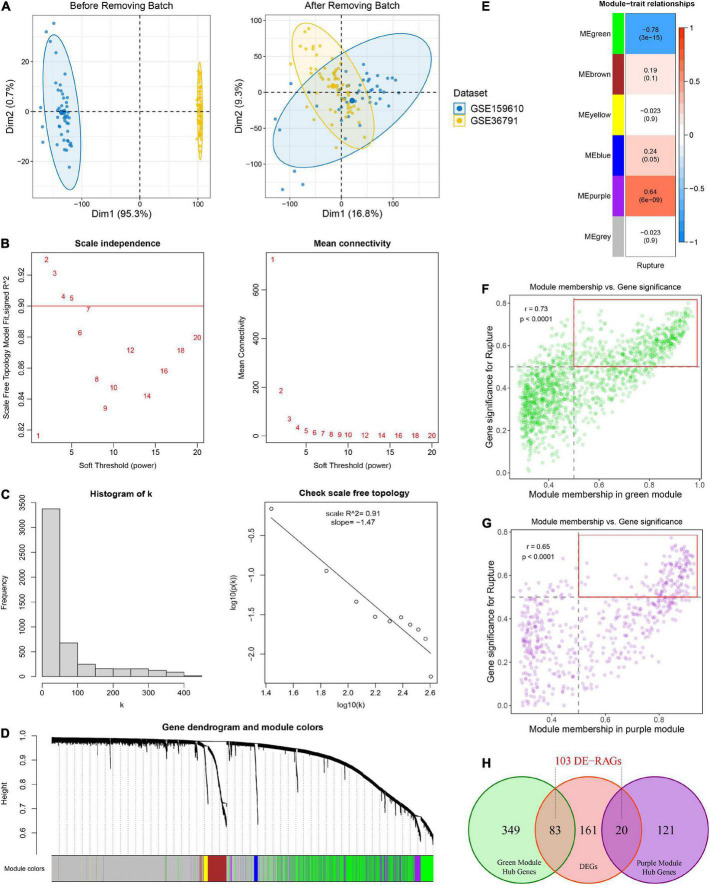
Identification of rupture-associated genes (RAGs) by WGCNA algorithm. **(A)** Principal component analysis before and after removing batch effects from non-biological technical biases. **(B)** Scale-free network analysis under different soft-thresholding powers. When the soft power of β ≥ 3, the scale-free *R*^2^ is greater than 0.9 and mean connectivity tends to be stable. **(C)** Histogram of connectivity distribution and checking the scale free topology when β = 3. **(D)** Gene hierarchical clustering under optimal soft-thresholding power. Genes with similar expression patterns were assigned to co-expression modules represented by the same color. **(E)** Heatmap of the correlation between module eigengenes and rupture status. Red and blue represent positive and negative correlations, respectively. **(F,G)** Correlation between GS and MM in the green **(F)** and purple **(G)** modules. Dots within the red rectangle were defined as hub RAGs, with both high GS and MM. **(H)** Venn diagram of intersection between hub RAGs and differentially expressed genes (DEGs).

The publicly available datasets GSE36791 and GSE159610 were integrated into one cohort (denoted as public cohort) after removing batch effects, and the principal component analysis before and after batch-correction was shown in Figure 2A. Only patients with RIAs or UIAs were included in this study. Ultimately, a total of 68 patients and 14070 genes were retained for subsequent analysis.

Weighted gene co-expression network analysis was performed on top 5,000 genes selected according to the median absolute deviation in the public cohort. The soft power of β = 3 (scale-free *R*^2^ = 0.91) was determined as an appropriate soft threshold to acquire gene co-expression modules (Figures 2B, C). Subsequently, the “blockwiseModules” function was utilized to carry out dynamic tree clipping and average hierarchical clustering (Figure 2D). As a consequence, a total of five modules were identified, indicating by green, brown, yellow, blue, and purple modules, respectively (Figures 2D, E). In addition, 2,607 out of 5,000 genes that failed to cluster into any module were shown as gray. The Eigen gene (first principal component of gene expression within a module) was considered as the representative of the module. Furthermore, the correlations between modules and rupture state were calculated. The purple module and the green module exhibited the highest positive and negative correlation with IA rupture, respectively (Figure 2E). About 141 of the 570 genes in the purple module and 432 of the 1442 genes in the green module displayed both high gene significance (>0.5) and module membership (>0.5), which were extracted as hub RAGs (Figures 2F, G).

### Machine learning-based integrative establishment of a rupture risk signature

**FIGURE 3:**
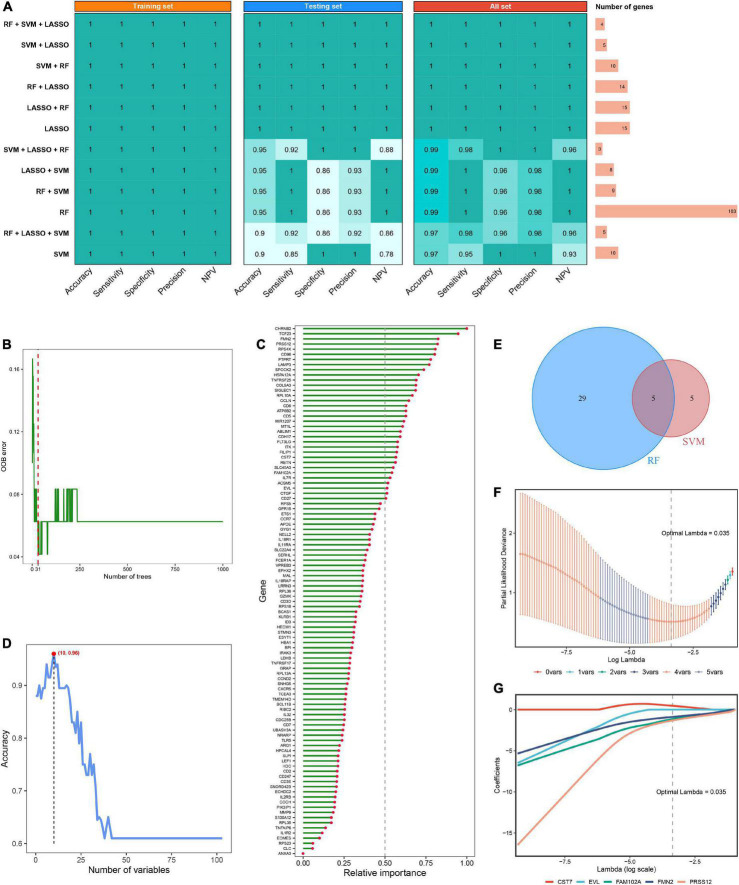
Machine learning-based integrative approaches for signature generation. **(A)** Accuracy, sensitivity, specificity, precision and NPV of 12 kinds of dichotomous models in the training set, validation set and all set. The rightmost panel displayed the number of constituent genes for each model. **(B)** Out of bag (OOB) error rate reached a minimum when the number of trees was equal to 31 in RF. **(C)** Relative importance of 103 initial input gene variables calculated in RF. Genes with relative importance greater than 0.5 were extracted as important gene variables. **(D)** Accuracy reached a maximum when the number of variables was equal to 10 in SVM. **(E)** Venn diagram of the intersection of important gene variables obtained from RF and SVM pre-screening. **(F)** The optimal lambda was determined when the partial likelihood deviance reached the minimum value in LASSO regression. **(G)** LASSO coefficient profiles of the candidate genes for MLDGS construction.

Using the *limma* R package, a total of 264 DEGs were identified between RIAs and UIAs. After taking the intersection with 573 hub RAGs, 103 differentially expressed RAGs were obtained as input feature variables for the MLDGS model construction (Figure 2H). According to our machine learning-based integrative procedure, 12 kinds of dichotomous models were fitted in the training set and further reproduced in the validation set and all set. The accuracy, sensitivity, specificity, precision and NPV of each model in the training set, validation set and all set, as well as the number of model genes, were shown in Figure 3A. Intriguingly, 6 kinds of models displayed excellent and equal average accuracy (1.00), but the RF + SVM + LASSO model contained the least number of model genes and was considered the optimal model. In detail, this optimal model was derived from the prior feature reduction by RF and SVM and the final modeling by LASSO regression. In the RF-fitted model, 34 out of 103 feature genes with relative importance greater than 0.5 were extracted as important gene variables (Figures 3B, C). In the SVM-fitted models, 10 model constituent genes were extracted as important feature genes (Figure 3D). These two important feature gene lists were taken to intersect, resulting in five crucial genes for the final LASSO regression modeling (Figure 3E). According to the LOOCV framework, the LASSO regression model reached an optimum when the lambda (λ) was equal to 0.035 (Figure 3F), containing four gene variables (Figure 3G). A risk score for each patient was calculated based on the relative expression of the four model genes weighted by their LASSO regression coefficients. The detailed formula as follow: MLDGS = 0.475*Exp (*CST7*) - 1.132*Exp (*FAM102A*) - 0.877*Exp (*FMN2*) -1.318*Exp (*PRSS12*) + 1.012; where the *Exp* represents the relative expression (z-score) of the corresponding gene.

#### Evaluation of the MLDGS model

**FIGURE 4:**
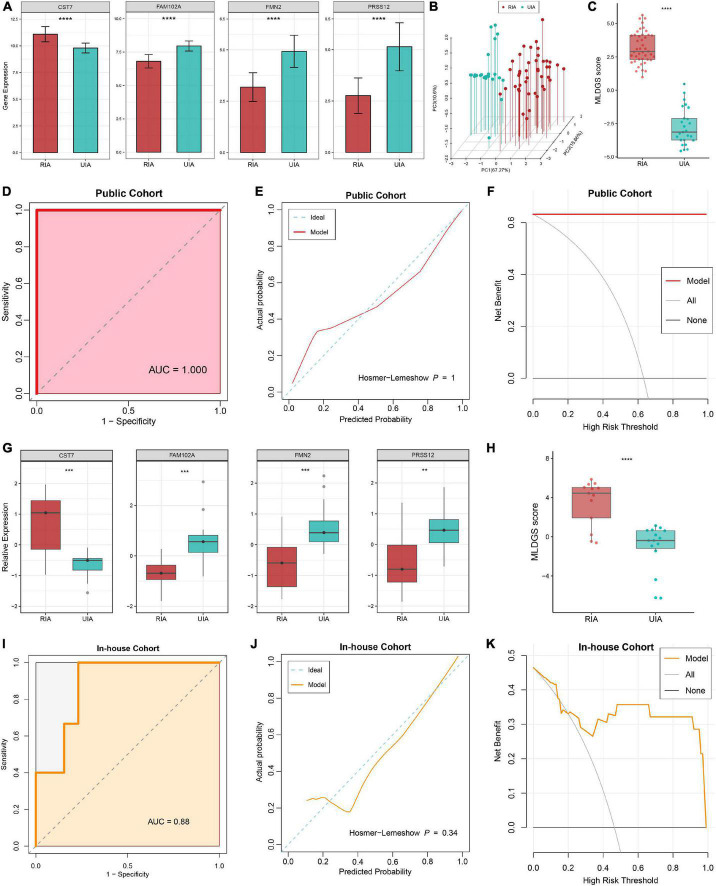
Evaluation and validation of the MLDGS model. **(A)** Differential expression of four model genes between RIA and UIA groups in public cohort. **(B)** Principal component analysis showed that RIAs and UIAs could be well distinguished based on the four model genes. **(C)** The distribution of MLDGS score between RIA and UIA groups in public cohort. D-F. ROC curve **(D)**, calibration curve **(E)** and decision curve **(F)** for the MLDGS to identify RIAs in public cohort. **(G)** Relative expression of four model genes between RIA and UIA groups in our in-house cohort. **(H)** The distribution of MLDGS score between RIA and UIA groups in our in-house cohort. **(I–K)** ROC curve **(I)**, calibration curve **(J)** and decision curve **(K)** for the MLDGS to identify RIAs in our in-house cohort. Statistic tests: two-sided *t*-test. ^ns^ *P* > 0.05, *P < 0.05,**P < 0.01, ***P < 0.001, and****P < 0.0001.

The expression differences of the four model genes between RIAs and UIAs were illustrated in Figure 4A. *CST7*, an immune regulation-relevant gene encoding cystatin F, was upregulated in patients with RIAs; whereas *FAM102A*, *FMN2* and *PRSS12* were downregulated in patients with RIAs. Principal component analysis showed that RIAs and UIAs could be well distinguished based on these gene variables (Figure 4B). According to our established MLDGS model, a risk score was calculated for each patient. Patients with RIAs presented a higher MLDGS scores compared to patients with UIAs (Figure 4C). Furthermore, ROC curve, calibration curve and DCA were performed to evaluate the performance of the model. The AUC of MLDGS for recognizing RIAs was equal to 1.00 (95% CI: 1.00–1.00), exhibiting excellent discrimination (Figure 4D). The calibration curve showed high agreement between model predictions and actual observations, and the Hosmer-Lemeshow goodness-of-fit test *P* = 1.00, suggesting an appreciable performance of the MLDGS model (Figure 4E). The DCA indicated that the MLDGS model exhibited a superior clinical net benefit than either the treat-all or treat-none strategy (Figure 4F). These data suggested that the MLDGS possessed an adequate performance for detecting the patients with high risk of IA rupture, which may be able to effectively optimize the clinical decision-making process for IA patients.

#### Validation in our in-house clinical cohort

To verify the performance and robustness of the model, we further detected the expression of the four model genes in an in-house clinical of 28 IA patients by qRT-PCR. Consistently, *CST7* was upregulated in patients with RIAs, while *FAM102A*, *FMN2* and *PRSS12* were downregulated in patients with RIAs (Figure 4G). The MLDGS score was next calculated for each patient, and the patients with RIAs still possessed higher risk scores than those with UIAs (Figure 4H). Furthermore, ROC curve, calibration curve and DCA were also performed in our in-house clinical cohort. The AUC of MLDGS for recognizing RIAs was equal to 0.88 (95% CI: 0.74–1.00; Figure 4I), the calibration curve showed an appreciable agreement between model predictions and actual observations (Hosmer-Lemeshow *P* = 0.34, Figure 4J), and DCA indicated that the MLDGS still retained an impressive net benefit in our in-house cohort (Figure 4K). Collectively, all these results supported our findings in the discovery cohort, which validated that our MLDGS model was quite accurate and robust for evaluating the rupture risk of IAs.

### The immune landscape and pathological features in peripheral circulation

**FIGURE 5:**
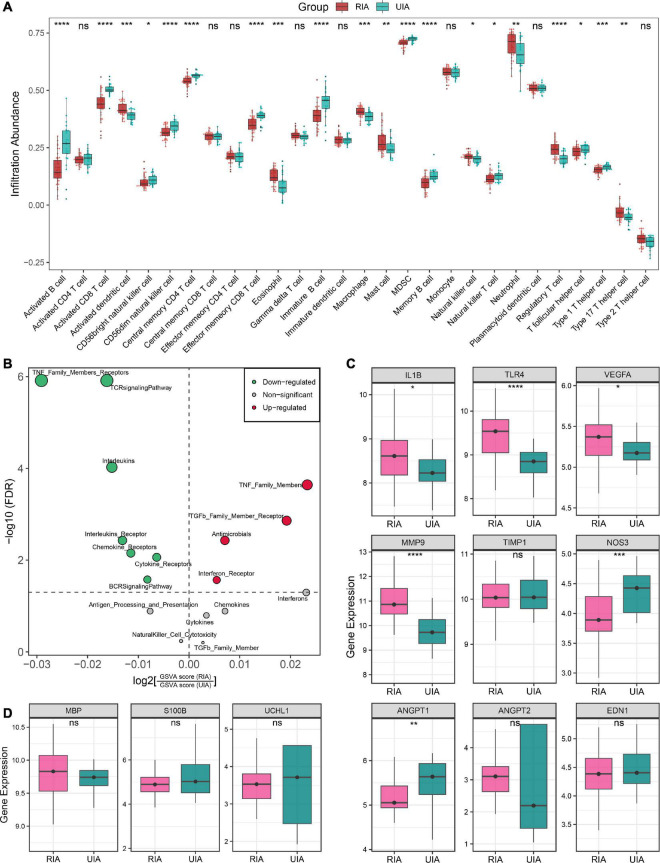
Immune landscape and pathological features of peripheral circulation in patients with RIAs and UIAs. **(A)** Relative infiltration abundance of 28 immune cell subtypes between RIA and UIA groups. **(B)** Volcano plot of differentially enriched immune-related gene sets. Red and green dots indicate globally up-regulated and down-regulated gene sets, respectively. **(C,D)** Relative expression of genes regarding vascular stability **(C)** and brain injury **(D)** between RIA and UIA groups in our in-house cohort. Statistic tests: two-sided *t*-test: ^ns^ *P* > 0.05, *P < 0.05, **P < 0.01, ***P < 0.001, and****P < 0.0001.

As illustrated in Figure 5A, the landscape of circulating immune cell infiltration between RIAs and UIAs was systemically revealed. Compared to the UIAs, the patients with RIAs exhibited a relatively higher abundance of innate immune cells such as activated dendritic cells, eosinophil, neutrophil and macrophage, and a relatively less abundance of lymphocytes such as activated B cell, immature B cell, memory B cell, CD8^+^ T cell, effector memory CD8^+^ T cell, central memory CD4^+^ T cell and Th1 cell. Intriguingly, CD56^+^ NK cell, CD56- NK cell, and NK T cell were reduced in RIA patients, and some immunosuppression lymphocytes such as regulatory T cell were synergistically elevated in RIAs. Moreover, GSVA analysis indicated that the overall enrichment levels of immune-related gene sets were also aberrant in peripheral circulation. TNF family, TGF-b receptor family, antimicrobials and interferon receptors were overall upregulated in RIAs, whereas TCR and BCR signaling pathway, interleukins, cytokines and chemokines were overall downregulated in RIAs (Figure 5B). Taken together, above results unveiled a complex and disturbed immune environment in peripheral circulation of IA patients.

Furthermore, we also investigated the gene expression of a series of previously reported molecules regarding vascular stability and brain injury (Figures 5C, D). In the RIA groups, inflammatory molecules *IL1B*, *TLR4* and *VEGFA*, and ECM metabolism-mediating molecule *MMP9* were significantly upregulated; *ANGPT2*, encoding angiopoietin-II that mediates vascular smooth muscle apoptosis, exhibited an upward trend in RIAs although the test *P* value was not significant enough; and the *ANGPT1*, encoding angiopoietin-I that protects vascular smooth muscle cells, was remarkably downregulated (Figure 5C). These data implied the enhanced intrinsic vascular instability in RIA patients, which may be one of the latent biological interpretations for the high risk of aneurysm rupture. Moreover, *NOS3*, encoding eNOS that mediates vasodilation, was significantly downregulated in RIAs, which implied a high risk of CVS onset (Figure 5C). *EDN1*, encoding the endothelin-1 that mediates vasoconstrictive, displayed no significantly change in the RIA group. Brain injury-relevant biomarkers *UCHL1*, *S100B*, and *MBP* were also investigated, but no significant difference was observed between RIA and UIA groups (Figure 5D). Collectively, patients with RIAs were characterized by high abundance of innate immune cells, less abundance of lymphocytes, immune gene family expression disorder, and enhanced vascular instability.

### Relationships between the MLDGS and immunopathological features

**FIGURE 6:**
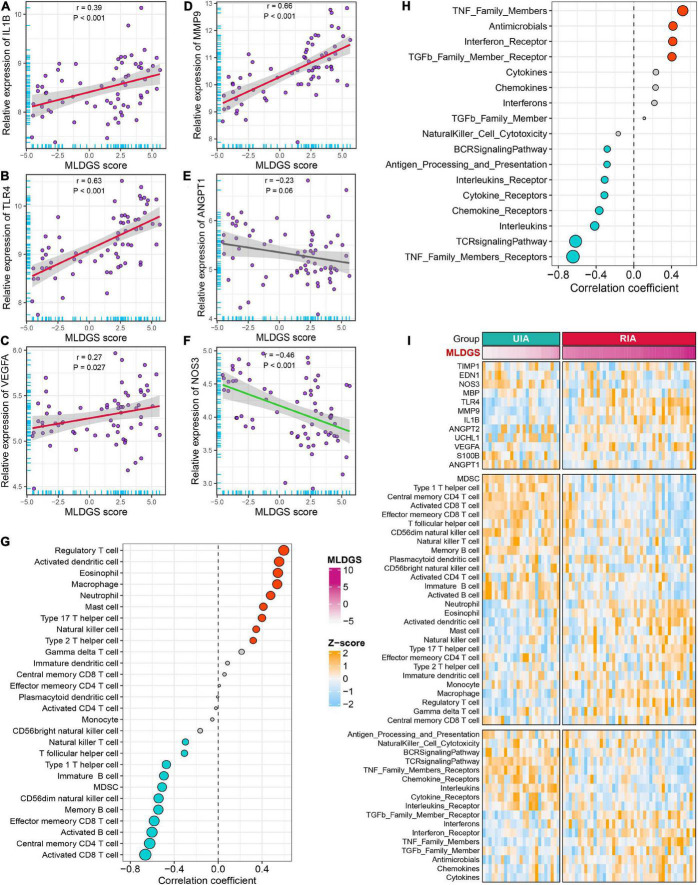
Relationships between the MLDGS and immunopathological features. **(A–F)** Correlation analysis of MLDGS score with the expression of *IL1B* **(A)**, *TLR4* **(B)**, *VEGFA* **(C)**, *MMP9* **(D)**, *ANGPT1* **(E)**, and *NOS3* **(F)**. **(G,H)** Relationship between MLDGS and immune cells and immune-related gene sets. Red and turquoise represent significant positive and negative correlations, respectively. **(I)** Global landscape of the relationship between MLDGS and various immunopathological features. Statistics: Pearson’s correlation; two-sided *t*-test.

To explore the link between the MLDGS and circulating immunological and pathological features, the corresponding correlation analysis was carried out. Similarly, the MLDGS score was mainly positively related to activated dendritic cell (*r* = 0.56, *P* < 0.001), eosinophil (*r* = 0.54, *P* < 0.001), neutrophil (*r* = 0.48, *P* < 0.001), macrophage (*r* = 0.54, *P* < 0.001) and mast cell (*r* = 0.41, *P* < 0.001), and negatively related to activated B cell (*r* = −0.60, *P* < 0.001), immature B cell (*r* = −0.49, *P* < 0.001), memory B cell (*r* = −0.54, P < 0.001), CD8^+^ T cell (*r* = −0.66, *P* < 0.001), effector memory CD8^+^ T cell (*r* = −0.58, *P* < 0.001), central memory CD4^+^ T cell (*r* = −0.62, *P* < 0.001), Th1 cell (*r* = −0.47, *P* < 0.001), CD56^–^ NK cell (*r* = −0.54, *P* < 0.001), and NK T cell (*r* = −0.30, *P* = 0.014; Figure 6G). Significant positive and negative associations were also observed between the MLDGS and upregulated and downregulated immune gene sets, respectively (Figure 6H). Moreover, the MLDGS was positively correlated with *IL1B* (*r* = 0.39, *P* = 0.001), *TLR4* (*r* = 0.63, *P* < 0.001), *VEGFA* (*r* = 0.27, *P* = 0.024), and *MMP9* (*r* = 0.66, *P* < 0.001; Figures 6A–D), and negatively correlated with *ANGPT1* (*r* = −0.23, *P* = 0.06) and *NOS3* (*r* = −0.46, *P* < 0.001; Figures 6E, F), although not significant enough in *ANGPT1*. Overall, higher MLDGS scores may represent higher abundance of innate immune cells, less abundance of adaptive immune cells, and worse immune and vascular instability relevant molecular features (Figure 6I), which implied a potential of MLDGS to assess the circulating immunopathological landscape and the risk of aneurysm rupture or CVS onset in patients with IAs.

### Potential biological mechanisms underlying the MLDGS

**FIGURE 7:**
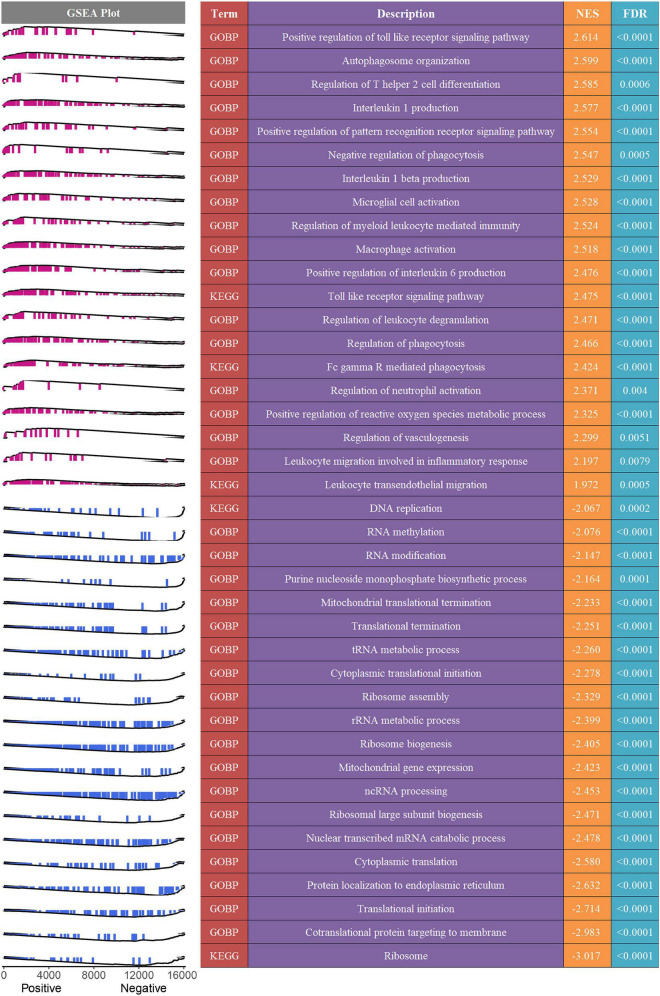
Potential biological mechanisms underlying the MLDGS. The right panel presented some important biological pathways that were significantly enriched (FDR < 0.01) in GSEA. NES > 0 and NES < 0 suggested pathway activation and inhibition, respectively. NES, normalized enrichment score. The left panel displayed a brief gene set enrichment map of these specific pathways and their relationship to the MLDGS. Purple and blue represented positive and negative correlation between pathway and MLDGS, respectively.

To decipher the latent biological mechanisms underlying the MLDGS, all genes were ranked in descending order based on their correlations with the MLDGS and subsequently subjected to the *clusterProfiler* R package for enrichment analysis. As illustrated in Figure 7, the MLDGS was positively correlated with numerous pathways related to immunomodulation and reactive oxygen metabolism, such as positive regulation of toll-like receptor signaling pathway, regulation of T helper 2 cell differentiation, positive regulation of pattern recognition receptor signaling pathway, interleukin 1 beta production, regulation of myeloid leukocyte mediated immunity, leukocyte migration involved in inflammatory response, leukocyte transendothelial migration, microglial cell activation, and positive regulation of reactive oxygen species metabolic process; whereas gene expression-associated biological pathways were negatively correlated with the MLDGS, such as ribosome pathway, co-translational protein targeting to membrane, translational initiation, cytoplasmic translation, nuclear transcribed mRNA catabolic process, ncRNA processing, and cytoplasmic translational initiation. These results suggested the potential biological mechanisms underlying the MLDGS, in which immunomodulatory and metabolic pathways were activated while transcription and translation processes were inhibited in patients with high MLDGS scores.

## Discussion

Intracranial aneurysm is an uncommon but severe subtype of cerebrovascular disease, with high mortality after aneurysm rupture. Endovascular or neurosurgical treatment is often considered to prevent aneurysm rupture, but a comprehensive assessment of disease status to determine the appropriate treatment demand and the choice of intervention opportunity is also needed in clinical setting. At present, the dynamic monitoring of some untreated aneurysms mostly relies on repeated magnetic resonance angiography or computed tomography angiography (38), without regard to the molecular pathological status. To perfect the precision diagnosis and treatment system of IA, developing available molecular detection techniques is imperative.

In the present study, WGCNA was utilized to identify vital rupture-related gene biomarkers in peripheral blood. With the expression profiles of these genes, we developed a machine learning-based integrative procedure to construct a MLDGS risk indicator. In total, 12 kinds of dichotomous models were fitted in the training set and were further reproduced in the validation set and the whole public cohort. The RF + SVM + LASSO combination with the highest average accuracy and the least number of model genes was selected as the optimal model. The advantage of modeling by machine learning algorithms and their combinations is that the combined application of algorithms can reduce the dimension of variables in advance and then more accurately fit the prediction model, making the model both accurate and simple. Several approaches encompassing ROC curve, calibration curve and DCA were employed to evaluate the model performance in public cohort, and all of them suggested that the MLDGS possessed an excellent accuracy for recognizing RIA patients from all populations with IAs. Moreover, independent validation in our in-house clinical cohort showed that MLDGS maintained adequate accuracy and stability for recognizing RIA patients, which suggested a strong robustness of the model. This result also indicated that the MLDGS can be reproduced using a simple qRT-PCR assay technology similar to the COVID-19 nucleic acid assay, making it inexpensive and convenient for clinical application.

The progression and rupture of intracranial aneurysms are driven by a variety of pathological factors, among which immune inflammation and vascular stability are of critical importance. Mastering these microscopic changes may facilitate a more precise assessment of pathological conditions and aneurysm risk. In the present study, we detected that the MLDGS was positively correlated with most of innate immune cells such as neutrophils, eosinophils, mast cells and macrophages, which are vital inflammatory cells mediating aneurysm wall damage and rupture (39–41). Conversely, MLDGS was negatively correlated with most of lymphocytes such as activated B cells, immature B cells, memory B cells, CD8^+^ T cells, effector memory CD8^+^ T cells, central memory CD4^+^ T cells, NK T cells and Th1 cells, implying suppressed peripheral adaptive immunity and increased risk of post-stroke infection (42) in patients with high MLDGS scores.

The molecular pathological status was not only related to the rupture risk but also associated with the complications of IA. Hence, the potential association between MLDGS and a series of previously reported molecules related to vascular stability was also investigated. As a consequence, MLDGS was remarkably positively correlated with *IL1B*, *TLR4*, *VEGFA* and *MMP9*, and negatively correlated with *NOS3*. Among these genes, *IL1B*, *TLR4*, *VEGFA*, as well as *MMP2*, are vital molecules mediating inflammatory injury and ECM metabolism in the vascular wall, respectively, which are closely related to the vascular instability and rupture risk of IA (1, 31–33). *NOS3*, encoding eNOS, participates in the regulation of vasodilator nitric oxide production (43). Vasodilatory dysfunction resulting from downregulation of eNOS expression is one of the crucial pathogeneses of CVS. Taken together, higher MLDGS score may represent higher expression of *IL1B*, *TLR4*, *VEGFA* and *MMP2*, and lower expression of *NOS3*, implying inferior vascular stability at the molecular level and high risk of aneurysm rupture and CVS onset.

Clinicians often confronted with a dilemma regarding the judgment of therapeutic demand of IAs. Indeed, several multigene signatures for unruptured intracranial aneurysms (UIAs) or ruptured intracranial aneurysms (RIAs) have been developed in recent years (44–46); however, they are all IA diagnostic models constructed using healthy subjects as controls and still without the in-depth assessment of molecular pathological features. In clinical settings, a biomarker with the ability to identify high-risk patients from the population with established IAs may be more appropriate for clinical decision-making. Therefore, the present study focused on finding gene biomarkers capable of distinguishing RIA from UIA in peripheral blood samples, and constructing a multigene model to make its diagnostic or predictive accuracy more robust. Overall, patients with high MLDGS scores may possess adverse biological alterations encompassing inflammatory activation, suppressed peripheral adaptive immunity, ECM hypermetabolism and vasodilatory dysfunction, and thus present with higher risk of IA rupture, post-stroke infection and CVS onset, requiring more vigilant attention or timely therapeutic intervention.

Although we preliminarily confirmed the excellent performance and transformation potential of the MLDGS model, certain limitations should also be acknowledged. First, both the public cohort for modeling and the in-house cohort for validation are small samples, and further prospective clinical verification of MLDGS with larger samples is required prior to clinical application. Second, the functions of the four model genes remain to be elucidated in IAs, and further *in vitro* and *in vivo* experiments are needed to reveal their roles.

## Conclusion

Using a multitude of bioinformatics and machine learning algorithms, the present study developed an accurate, stable and pleiotropic gene signature for assessing rupture risk and circulating immunopathologic landscape in patients with IAs. This MLDGS model can serve as a beneficial and convenient molecular assay tool to optimize the IA risk surveillance system and assist clinical decision-making, contributing to advances in IA precision medicine.

## Data availability statement

The original contributions presented in this study are included in the article/Supplementary material, further inquiries can be directed to the corresponding authors.

## Ethics statement

The studies involving human participants were reviewed and approved by Ethical Committee of Zhengzhou University People’s Hospital. The patients/participants provided their written informed consent to participate in this study.

## Author contributions

TL and YKH designed this work. TL and ZL integrated and analyzed the data. YYH, TL, CM, YL, and RJ performed the experiments. TL wrote this manuscript. YKH, TL, TYL, ZL, YYH, SC, LD, DG, and CG edited and revised the manuscript. All authors contributed to the article and approved the submitted version.

## Footnotes

1
http://www.ncbi.nlm.nih.gov/geo/

2
https://www.immport.org/
