# Supplementary material for: A machine learning-derived gene signature for assessing rupture risk and circulatory immunopathologic landscape in patients with intracranial aneurysms
Source: Front Cardiovasc Med. 2023 Feb 10;10:1075584. doi: 10.3389/fcvm.2023.1075584 (PMC9950511; doi:10.3389/fcvm.2023.1075584)
Supplement: Supplementary file 1 [file Data_Sheet_1.PDF]

Supplemental Tables

Table S1. Baseline information for two public datasets and one in-house clinical cohort.

Table S2. The forward and reverse primers for qRT-PCR.

Table S3. The gene sets for marking 28 immune cell types.

Table S4. 17 immune-related gene sets collected from the Immport.

Table S1. Baseline information for two public datasets and one in-house clinical cohort.

Cohort	GSE36791	GSE159610	In-house
**Platform**	GPL10558	GPL16791	-
**Data Type**	Microarray	RNA-seq	qRT-PCR
**N**	61	47	28
**Group**			
RIA	43	0	13
UIA	0	25	15
IA-free	18	22	0
**Male/female**	27/34	-	10/18
**Age (years)***	-	-	56.0 (53.8, 64.0)

RIA, ruptured intracranial aneurysm; UIA, unruptured intracranial aneurysm.

*Data are median (Q_1, Q_3).

Table S2. The forward and reverse primers for qRT-PCR.

Genes	Forward (5' to 3')	Reverse (5' to 3')
GAPDH	AGCTCACTGGCATGGCCTTC	CGCCTGCTTCACCACCTTCT
CST7	GTGTGAAGCCAGGATTTCCTAA	TGTCGTTCGTGCAGTTGTTGA
FAM102A	TCCCGCAACTCCAGCTATG	CTTCCGCCTGAAAGAGCGA
FMN2	CAGAGCCTCGGTGTTTTCCAA	GCACACTCGGTATCCGACAG
PRSS12	GCTTGGCTACAAGGGTCCTG	CGGCAGTTGTGTCTTCCAAT

Table S3. The gene sets for marking 28 immune cell types.

**Gene**	**Cell.type**	**Category**
ADAM28	Activated B cell	Adaptive
CD180	Activated B cell	Adaptive
CD79B	Activated B cell	Adaptive
BLK	Activated B cell	Adaptive
CD19	Activated B cell	Adaptive
MS4A1	Activated B cell	Adaptive
TNFRSF17	Activated B cell	Adaptive
IGHM	Activated B cell	Adaptive
GNG7	Activated B cell	Adaptive
MICAL3	Activated B cell	Adaptive
SPIB	Activated B cell	Adaptive
HLA-DOB	Activated B cell	Adaptive
IGKC	Activated B cell	Adaptive
PNOC	Activated B cell	Adaptive
FCRL2	Activated B cell	Adaptive
BACH2	Activated B cell	Adaptive
CR2	Activated B cell	Adaptive
TCL1A	Activated B cell	Adaptive
AKNA	Activated B cell	Adaptive
ARHGAP25	Activated B cell	Adaptive
CCL21	Activated B cell	Adaptive
CD27	Activated B cell	Adaptive
CD38	Activated B cell	Adaptive
CLEC17A	Activated B cell	Adaptive
CLEC9A	Activated B cell	Adaptive
CLECL1	Activated B cell	Adaptive
AIM2	Activated CD4 T cell	Adaptive
BIRC3	Activated CD4 T cell	Adaptive
BRIP1	Activated CD4 T cell	Adaptive
CCL20	Activated CD4 T cell	Adaptive
CCL4	Activated CD4 T cell	Adaptive
CCL5	Activated CD4 T cell	Adaptive
CCNB1	Activated CD4 T cell	Adaptive
CCR7	Activated CD4 T cell	Adaptive
DUSP2	Activated CD4 T cell	Adaptive
ESCO2	Activated CD4 T cell	Adaptive
ETS1	Activated CD4 T cell	Adaptive
EXO1	Activated CD4 T cell	Adaptive
EXOC6	Activated CD4 T cell	Adaptive
IARS	Activated CD4 T cell	Adaptive
ITK	Activated CD4 T cell	Adaptive

KIF11	Activated CD4 T cell	Adaptive
KNTC1	Activated CD4 T cell	Adaptive
NUF2	Activated CD4 T cell	Adaptive
PRC1	Activated CD4 T cell	Adaptive
PSAT1	Activated CD4 T cell	Adaptive
RGS1	Activated CD4 T cell	Adaptive
RTKN2	Activated CD4 T cell	Adaptive
SAMSN1	Activated CD4 T cell	Adaptive
SELL	Activated CD4 T cell	Adaptive
TRAT1	Activated CD4 T cell	Adaptive
ADRM1	Activated CD8 T cell	Adaptive
AHSA1	Activated CD8 T cell	Adaptive
C1GALT1C1	Activated CD8 T cell	Adaptive
CCT6B	Activated CD8 T cell	Adaptive
CD37	Activated CD8 T cell	Adaptive
CD3D	Activated CD8 T cell	Adaptive
CD3E	Activated CD8 T cell	Adaptive
CD3G	Activated CD8 T cell	Adaptive
CD69	Activated CD8 T cell	Adaptive
CD8A	Activated CD8 T cell	Adaptive
CETN3	Activated CD8 T cell	Adaptive
CSE1L	Activated CD8 T cell	Adaptive
GEMIN6	Activated CD8 T cell	Adaptive
GNLY	Activated CD8 T cell	Adaptive
GPT2	Activated CD8 T cell	Adaptive
GZMA	Activated CD8 T cell	Adaptive
GZMH	Activated CD8 T cell	Adaptive
GZMK	Activated CD8 T cell	Adaptive
IL2RB	Activated CD8 T cell	Adaptive
LCK	Activated CD8 T cell	Adaptive
MPZL1	Activated CD8 T cell	Adaptive
NKG7	Activated CD8 T cell	Adaptive
PIK3IP1	Activated CD8 T cell	Adaptive
PTRH2	Activated CD8 T cell	Adaptive
TIMM13	Activated CD8 T cell	Adaptive
ZAP70	Activated CD8 T cell	Adaptive
ABHD3	Central memory CD4 T cell	Adaptive
AHNAK	Central memory CD4 T cell	Adaptive
ANXA2P2	Central memory CD4 T cell	Adaptive
AQP3	Central memory CD4 T cell	Adaptive
ATHL1	Central memory CD4 T cell	Adaptive
BMI1	Central memory CD4 T cell	Adaptive
BZW2	Central memory CD4 T cell	Adaptive
CD63	Central memory CD4 T cell	Adaptive

COL4A1	Central memory CD4 T cell	Adaptive
CYLD	Central memory CD4 T cell	Adaptive
ELMO2	Central memory CD4 T cell	Adaptive
FYN	Central memory CD4 T cell	Adaptive
GLIPR1	Central memory CD4 T cell	Adaptive
GSS	Central memory CD4 T cell	Adaptive
IFITM2	Central memory CD4 T cell	Adaptive
ITGB1	Central memory CD4 T cell	Adaptive
ITGB2	Central memory CD4 T cell	Adaptive
KLF5	Central memory CD4 T cell	Adaptive
LSP1	Central memory CD4 T cell	Adaptive
NDUFB9	Central memory CD4 T cell	Adaptive
PKM2	Central memory CD4 T cell	Adaptive
SFXN3	Central memory CD4 T cell	Adaptive
SIRPG	Central memory CD4 T cell	Adaptive
SMAD4	Central memory CD4 T cell	Adaptive
STX4	Central memory CD4 T cell	Adaptive
TRADD	Central memory CD4 T cell	Adaptive
VIM	Central memory CD4 T cell	Adaptive
XRCC6	Central memory CD4 T cell	Adaptive
ACTN4	Central memory CD8 T cell	Adaptive
ADAM12	Central memory CD8 T cell	Adaptive
ADCY9	Central memory CD8 T cell	Adaptive
F13A1	Central memory CD8 T cell	Adaptive
FCER1G	Central memory CD8 T cell	Adaptive
FCGR3B	Central memory CD8 T cell	Adaptive
FGF7	Central memory CD8 T cell	Adaptive
FKBP4	Central memory CD8 T cell	Adaptive
GLUD1	Central memory CD8 T cell	Adaptive
GM2A	Central memory CD8 T cell	Adaptive
GUSB	Central memory CD8 T cell	Adaptive
IL1RN	Central memory CD8 T cell	Adaptive
NOL11	Central memory CD8 T cell	Adaptive
NTRK1	Central memory CD8 T cell	Adaptive
RARA	Central memory CD8 T cell	Adaptive
RNF128	Central memory CD8 T cell	Adaptive
SIGLEC1	Central memory CD8 T cell	Adaptive
TNFRSF11A	Central memory CD8 T cell	Adaptive
TOX4	Central memory CD8 T cell	Adaptive
UBA52	Central memory CD8 T cell	Adaptive
ULBP1	Central memory CD8 T cell	Adaptive
ATM	Effector memeory CD4 T cell	Adaptive
CASP3	Effector memeory CD4 T cell	Adaptive
CASQ1	Effector memeory CD4 T cell	Adaptive

CD300E	Effector memeory CD4 T cell	Adaptive
DARS	Effector memeory CD4 T cell	Adaptive
DOCK9	Effector memeory CD4 T cell	Adaptive
EXOSC9	Effector memeory CD4 T cell	Adaptive
EZH2	Effector memeory CD4 T cell	Adaptive
GDE1	Effector memeory CD4 T cell	Adaptive
IL34	Effector memeory CD4 T cell	Adaptive
NCOA4	Effector memeory CD4 T cell	Adaptive
NEFL	Effector memeory CD4 T cell	Adaptive
PDGFRL	Effector memeory CD4 T cell	Adaptive
PTGS1	Effector memeory CD4 T cell	Adaptive
REPS1	Effector memeory CD4 T cell	Adaptive
SCG2	Effector memeory CD4 T cell	Adaptive
SDPR	Effector memeory CD4 T cell	Adaptive
SIGLEC14	Effector memeory CD4 T cell	Adaptive
SIGLEC6	Effector memeory CD4 T cell	Adaptive
TAL1	Effector memeory CD4 T cell	Adaptive
TFEC	Effector memeory CD4 T cell	Adaptive
TIPIN	Effector memeory CD4 T cell	Adaptive
TPK1	Effector memeory CD4 T cell	Adaptive
UQCRB	Effector memeory CD4 T cell	Adaptive
USP9Y	Effector memeory CD4 T cell	Adaptive
WIPF1	Effector memeory CD4 T cell	Adaptive
ZCRB1	Effector memeory CD4 T cell	Adaptive
ACAP1	Effector memeory CD8 T cell	Adaptive
APOL3	Effector memeory CD8 T cell	Adaptive
ARHGAP10	Effector memeory CD8 T cell	Adaptive
ATP10D	Effector memeory CD8 T cell	Adaptive
C3AR1	Effector memeory CD8 T cell	Adaptive
CCR5	Effector memeory CD8 T cell	Adaptive
CD160	Effector memeory CD8 T cell	Adaptive
CD55	Effector memeory CD8 T cell	Adaptive
CFLAR	Effector memeory CD8 T cell	Adaptive
CMKLR1	Effector memeory CD8 T cell	Adaptive
DAPP1	Effector memeory CD8 T cell	Adaptive
FCRL6	Effector memeory CD8 T cell	Adaptive
FLT3LG	Effector memeory CD8 T cell	Adaptive
GZMM	Effector memeory CD8 T cell	Adaptive
HAPLN3	Effector memeory CD8 T cell	Adaptive
HLA-DMB	Effector memeory CD8 T cell	Adaptive
HLA-DPA1	Effector memeory CD8 T cell	Adaptive
HLA-DPB1	Effector memeory CD8 T cell	Adaptive
IFI16	Effector memeory CD8 T cell	Adaptive
LIME1	Effector memeory CD8 T cell	Adaptive

LTK	Effector memeory CD8 T cell	Adaptive
NFKBIA	Effector memeory CD8 T cell	Adaptive
SETD7	Effector memeory CD8 T cell	Adaptive
SIK1	Effector memeory CD8 T cell	Adaptive
TRIB2	Effector memeory CD8 T cell	Adaptive
ACP5	Gamma delta T cell	Adaptive
AQP9	Gamma delta T cell	Adaptive
BTN3A2	Gamma delta T cell	Adaptive
C1orf54	Gamma delta T cell	Adaptive
CARD8	Gamma delta T cell	Adaptive
CCL18	Gamma delta T cell	Adaptive
CD209	Gamma delta T cell	Adaptive
CD33	Gamma delta T cell	Adaptive
CD36	Gamma delta T cell	Adaptive
CDK5	Gamma delta T cell	Adaptive
IL10RB	Gamma delta T cell	Adaptive
KLRF1	Gamma delta T cell	Adaptive
LGALS1	Gamma delta T cell	Adaptive
MAPK7	Gamma delta T cell	Adaptive
KLHL7	Gamma delta T cell	Adaptive
KRT80	Gamma delta T cell	Adaptive
LAMC1	Gamma delta T cell	Adaptive
LCORL	Gamma delta T cell	Adaptive
LMNB1	Gamma delta T cell	Adaptive
MEIS3P1	Gamma delta T cell	Adaptive
MPL	Gamma delta T cell	Adaptive
FABP1	Gamma delta T cell	Adaptive
FABP5	Gamma delta T cell	Adaptive
FADD	Gamma delta T cell	Adaptive
MFAP3L	Gamma delta T cell	Adaptive
MINPP1	Gamma delta T cell	Adaptive
RPS24	Gamma delta T cell	Adaptive
RPS7	Gamma delta T cell	Adaptive
RPS9	Gamma delta T cell	Adaptive
DBNL	Gamma delta T cell	Adaptive
CCL13	Gamma delta T cell	Adaptive
CD22	Immature B cell	Adaptive
CYBB	Immature B cell	Adaptive
FAM129C	Immature B cell	Adaptive
FCRL1	Immature B cell	Adaptive
FCRL3	Immature B cell	Adaptive
FCRL5	Immature B cell	Adaptive
FCRLA	Immature B cell	Adaptive
HDAC9	Immature B cell	Adaptive

HLA-DQA1	Immature B cell	Adaptive
HVCN1	Immature B cell	Adaptive
KIAA0226	Immature B cell	Adaptive
NCF1	Immature B cell	Adaptive
NCF1B	Immature B cell	Adaptive
P2RY10	Immature B cell	Adaptive
SP100	Immature B cell	Adaptive
TXNIP	Immature B cell	Adaptive
STAP1	Immature B cell	Adaptive
TAGAP	Immature B cell	Adaptive
ZCCHC2	Immature B cell	Adaptive
AICDA	Memory B cell	Adaptive
CCNA2	Memory B cell	Adaptive
CDKN3	Memory B cell	Adaptive
CLCN5	Memory B cell	Adaptive
ENPP1	Memory B cell	Adaptive
FCER1A	Memory B cell	Adaptive
FCRL4	Memory B cell	Adaptive
MYC	Memory B cell	Adaptive
RUNX2	Memory B cell	Adaptive
SORL1	Memory B cell	Adaptive
SOX5	Memory B cell	Adaptive
STAT5A	Memory B cell	Adaptive
STAT5B	Memory B cell	Adaptive
TLR9	Memory B cell	Adaptive
CCL3L1	Regulatory T cell	Adaptive
CD72	Regulatory T cell	Adaptive
CLEC5A	Regulatory T cell	Adaptive
FOXP3	Regulatory T cell	Adaptive
ITGA4	Regulatory T cell	Adaptive
L1CAM	Regulatory T cell	Adaptive
LIPA	Regulatory T cell	Adaptive
LRP1	Regulatory T cell	Adaptive
LRRC42	Regulatory T cell	Adaptive
MARCO	Regulatory T cell	Adaptive
MMP12	Regulatory T cell	Adaptive
MNDA	Regulatory T cell	Adaptive
MRC1	Regulatory T cell	Adaptive
MS4A6A	Regulatory T cell	Adaptive
PELO	Regulatory T cell	Adaptive
PLEK	Regulatory T cell	Adaptive
PRSS23	Regulatory T cell	Adaptive
PTGIR	Regulatory T cell	Adaptive
ST8SIA4	Regulatory T cell	Adaptive

STAB1	Regulatory T cell	Adaptive
B3GAT1	T follicular helper cell	Adaptive
CDK5R1	T follicular helper cell	Adaptive
PDCD1	T follicular helper cell	Adaptive
BCL6	T follicular helper cell	Adaptive
CD200	T follicular helper cell	Adaptive
CD83	T follicular helper cell	Adaptive
CD84	T follicular helper cell	Adaptive
FGF2	T follicular helper cell	Adaptive
GPR18	T follicular helper cell	Adaptive
CEBPA	T follicular helper cell	Adaptive
CECR1	T follicular helper cell	Adaptive
CLEC10A	T follicular helper cell	Adaptive
CLEC4A	T follicular helper cell	Adaptive
CSF1R	T follicular helper cell	Adaptive
CTSS	T follicular helper cell	Adaptive
DMN	T follicular helper cell	Adaptive
DPP4	T follicular helper cell	Adaptive
LRRC32	T follicular helper cell	Adaptive
MC5R	T follicular helper cell	Adaptive
MICA	T follicular helper cell	Adaptive
NCAM1	T follicular helper cell	Adaptive
NCR2	T follicular helper cell	Adaptive
NRP1	T follicular helper cell	Adaptive
PDCD1LG2	T follicular helper cell	Adaptive
PDCD6	T follicular helper cell	Adaptive
PRDX1	T follicular helper cell	Adaptive
RAE1	T follicular helper cell	Adaptive
RAET1E	T follicular helper cell	Adaptive
SIGLEC7	T follicular helper cell	Adaptive
SIGLEC9	T follicular helper cell	Adaptive
TYRO3	T follicular helper cell	Adaptive
CHST12	T follicular helper cell	Adaptive
CLIC3	T follicular helper cell	Adaptive
IVNS1ABP	T follicular helper cell	Adaptive
KIR2DL2	T follicular helper cell	Adaptive
LGMN	T follicular helper cell	Adaptive
CD70	Type 1 T helper cell	Adaptive
TBX21	Type 1 T helper cell	Adaptive
ADAM8	Type 1 T helper cell	Adaptive
AHCYL2	Type 1 T helper cell	Adaptive
ALCAM	Type 1 T helper cell	Adaptive
B3GALNT1	Type 1 T helper cell	Adaptive
BBS12	Type 1 T helper cell	Adaptive

BST1	Type 1 T helper cell	Adaptive
CD151	Type 1 T helper cell	Adaptive
CD47	Type 1 T helper cell	Adaptive
CD48	Type 1 T helper cell	Adaptive
CD52	Type 1 T helper cell	Adaptive
CD53	Type 1 T helper cell	Adaptive
CD59	Type 1 T helper cell	Adaptive
CD6	Type 1 T helper cell	Adaptive
CD68	Type 1 T helper cell	Adaptive
CD7	Type 1 T helper cell	Adaptive
CD96	Type 1 T helper cell	Adaptive
CFHR3	Type 1 T helper cell	Adaptive
CHRM3	Type 1 T helper cell	Adaptive
CLEC7A	Type 1 T helper cell	Adaptive
COL23A1	Type 1 T helper cell	Adaptive
COL4A4	Type 1 T helper cell	Adaptive
COL5A3	Type 1 T helper cell	Adaptive
DAB1	Type 1 T helper cell	Adaptive
DLEU7	Type 1 T helper cell	Adaptive
DOC2B	Type 1 T helper cell	Adaptive
EMP1	Type 1 T helper cell	Adaptive
F12	Type 1 T helper cell	Adaptive
FURIN	Type 1 T helper cell	Adaptive
GAB3	Type 1 T helper cell	Adaptive
GATM	Type 1 T helper cell	Adaptive
GFPT2	Type 1 T helper cell	Adaptive
GPR25	Type 1 T helper cell	Adaptive
GREM2	Type 1 T helper cell	Adaptive
HAVCR1	Type 1 T helper cell	Adaptive
HSD11B1	Type 1 T helper cell	Adaptive
HUNK	Type 1 T helper cell	Adaptive
IGF2	Type 1 T helper cell	Adaptive
RCSD1	Type 1 T helper cell	Adaptive
RYR1	Type 1 T helper cell	Adaptive
SAV1	Type 1 T helper cell	Adaptive
SELE	Type 1 T helper cell	Adaptive
SELP	Type 1 T helper cell	Adaptive
SH3KBP1	Type 1 T helper cell	Adaptive
SIT1	Type 1 T helper cell	Adaptive
SLC35B3	Type 1 T helper cell	Adaptive
SIGLEC10	Type 1 T helper cell	Adaptive
SKAP1	Type 1 T helper cell	Adaptive
THUMPD2	Type 1 T helper cell	Adaptive
TIGIT	Type 1 T helper cell	Adaptive

ZEB2	Type 1 T helper cell	Adaptive
ENC1	Type 1 T helper cell	Adaptive
FAM134B	Type 1 T helper cell	Adaptive
FBXO30	Type 1 T helper cell	Adaptive
FCGR2C	Type 1 T helper cell	Adaptive
STAC	Type 1 T helper cell	Adaptive
LTC4S	Type 1 T helper cell	Adaptive
MAN1B1	Type 1 T helper cell	Adaptive
MDH1	Type 1 T helper cell	Adaptive
MMD	Type 1 T helper cell	Adaptive
RGS16	Type 1 T helper cell	Adaptive
IL12A	Type 1 T helper cell	Adaptive
P2RX5	Type 1 T helper cell	Adaptive
CD97	Type 1 T helper cell	Adaptive
ITGB4	Type 1 T helper cell	Adaptive
ICAM3	Type 1 T helper cell	Adaptive
METRNL	Type 1 T helper cell	Adaptive
TNFRSF1A	Type 1 T helper cell	Adaptive
IRF1	Type 1 T helper cell	Adaptive
HTR2B	Type 1 T helper cell	Adaptive
CALD1	Type 1 T helper cell	Adaptive
MOCOS	Type 1 T helper cell	Adaptive
TRAF3IP2	Type 1 T helper cell	Adaptive
TLR8	Type 1 T helper cell	Adaptive
TRAF1	Type 1 T helper cell	Adaptive
DUSP14	Type 1 T helper cell	Adaptive
IL17A	Type 17 T helper cell	Adaptive
IL17RA	Type 17 T helper cell	Adaptive
C2CD4A	Type 17 T helper cell	Adaptive
C2CD4B	Type 17 T helper cell	Adaptive
CA2	Type 17 T helper cell	Adaptive
CCDC65	Type 17 T helper cell	Adaptive
CEACAM3	Type 17 T helper cell	Adaptive
IL17C	Type 17 T helper cell	Adaptive
IL17F	Type 17 T helper cell	Adaptive
IL17RC	Type 17 T helper cell	Adaptive
IL17RE	Type 17 T helper cell	Adaptive
IL23A	Type 17 T helper cell	Adaptive
ILDR1	Type 17 T helper cell	Adaptive
LONRF3	Type 17 T helper cell	Adaptive
SH2D6	Type 17 T helper cell	Adaptive
TNIP2	Type 17 T helper cell	Adaptive
ABCA1	Type 17 T helper cell	Adaptive
ABCB1	Type 17 T helper cell	Adaptive

ADAMTS12	Type 17 T helper cell	Adaptive
ANK1	Type 17 T helper cell	Adaptive
ANKRD22	Type 17 T helper cell	Adaptive
B3GALT2	Type 17 T helper cell	Adaptive
CAMTA1	Type 17 T helper cell	Adaptive
CCR9	Type 17 T helper cell	Adaptive
CD40	Type 17 T helper cell	Adaptive
GPR44	Type 17 T helper cell	Adaptive
IFT80	Type 17 T helper cell	Adaptive
ASB2	Type 2 T helper cell	Adaptive
CSRP2	Type 2 T helper cell	Adaptive
DAPK1	Type 2 T helper cell	Adaptive
DLC1	Type 2 T helper cell	Adaptive
DNAJC12	Type 2 T helper cell	Adaptive
DUSP6	Type 2 T helper cell	Adaptive
GNAI1	Type 2 T helper cell	Adaptive
LAMP3	Type 2 T helper cell	Adaptive
NRP2	Type 2 T helper cell	Adaptive
OSBPL1A	Type 2 T helper cell	Adaptive
PDE4B	Type 2 T helper cell	Adaptive
PHLDA1	Type 2 T helper cell	Adaptive
PLA2G4A	Type 2 T helper cell	Adaptive
RAB27B	Type 2 T helper cell	Adaptive
RBMS3	Type 2 T helper cell	Adaptive
RNF125	Type 2 T helper cell	Adaptive
TMPRSS3	Type 2 T helper cell	Adaptive
GATA3	Type 2 T helper cell	Adaptive
BIRC5	Type 2 T helper cell	Adaptive
CDC25C	Type 2 T helper cell	Adaptive
CDC7	Type 2 T helper cell	Adaptive
CENPF	Type 2 T helper cell	Adaptive
CXCR6	Type 2 T helper cell	Adaptive
DHFR	Type 2 T helper cell	Adaptive
EVI5	Type 2 T helper cell	Adaptive
GSTA4	Type 2 T helper cell	Adaptive
HELLS	Type 2 T helper cell	Adaptive
IL26	Type 2 T helper cell	Adaptive
LAIR2	Type 2 T helper cell	Adaptive
ABCD1	Activated dendritic cell	Innate
C1QC	Activated dendritic cell	Innate
CAPG	Activated dendritic cell	Innate
CCL3L3	Activated dendritic cell	Innate
CD207	Activated dendritic cell	Innate
CD302	Activated dendritic cell	Innate

ATP5B	Activated dendritic cell	Innate
ATP5L	Activated dendritic cell	Innate
ATP6V1A	Activated dendritic cell	Innate
BCL2L1	Activated dendritic cell	Innate
C1QB	Activated dendritic cell	Innate
SNURF	Activated dendritic cell	Innate
SPCS3	Activated dendritic cell	Innate
CCNA1	Activated dendritic cell	Innate
CEACAM8	Activated dendritic cell	Innate
NOS2	Activated dendritic cell	Innate
SRA1	Activated dendritic cell	Innate
TNFRSF6B	Activated dendritic cell	Innate
TREM1	Activated dendritic cell	Innate
TREML1	Activated dendritic cell	Innate
RHOA	Activated dendritic cell	Innate
SLC25A37	Activated dendritic cell	Innate
TNFSF14	Activated dendritic cell	Innate
TREML4	Activated dendritic cell	Innate
VNN2	Activated dendritic cell	Innate
XPO6	Activated dendritic cell	Innate
CLEC4C	Activated dendritic cell	Innate
TNFAIP2	Activated dendritic cell	Innate
UBD	Activated dendritic cell	Innate
ACTR3	Activated dendritic cell	Innate
RAB1A	Activated dendritic cell	Innate
SLA	Activated dendritic cell	Innate
HLA-DQA2	Activated dendritic cell	Innate
SIGLEC5	Activated dendritic cell	Innate
SLAMF9	Activated dendritic cell	Innate
ABAT	CD56bright natural killer cell	Innate
C11orf75	CD56bright natural killer cell	Innate
C5orf15	CD56bright natural killer cell	Innate
CDHR1	CD56bright natural killer cell	Innate
DCAF12	CD56bright natural killer cell	Innate
DYNLL1	CD56bright natural killer cell	Innate
GPR137B	CD56bright natural killer cell	Innate
HCP5	CD56bright natural killer cell	Innate
HDGFRP2	CD56bright natural killer cell	Innate
KRT86	CD56bright natural killer cell	Innate
MLST8	CD56bright natural killer cell	Innate
ELMOD3	CD56bright natural killer cell	Innate
ENTPD5	CD56bright natural killer cell	Innate
FAM119A	CD56bright natural killer cell	Innate
FAM179A	CD56bright natural killer cell	Innate

CLIC2	CD56bright natural killer cell	Innate
COX7A2L	CD56bright natural killer cell	Innate
CREB3L4	CD56bright natural killer cell	Innate
CSF1	CD56bright natural killer cell	Innate
CSNK2A2	CD56bright natural killer cell	Innate
CSTA	CD56bright natural killer cell	Innate
CSTB	CD56bright natural killer cell	Innate
CTPS	CD56bright natural killer cell	Innate
CTSD	CD56bright natural killer cell	Innate
FST	CD56bright natural killer cell	Innate
GATA2	CD56bright natural killer cell	Innate
GMPR	CD56bright natural killer cell	Innate
HDC	CD56bright natural killer cell	Innate
HEY1	CD56bright natural killer cell	Innate
HOXA1	CD56bright natural killer cell	Innate
HS2ST1	CD56bright natural killer cell	Innate
HS3ST1	CD56bright natural killer cell	Innate
BCL11B	CD56bright natural killer cell	Innate
CDH3	CD56bright natural killer cell	Innate
MYL6B	CD56bright natural killer cell	Innate
NAA16	CD56bright natural killer cell	Innate
ClQA	CD56bright natural killer cell	Innate
ClQB	CD56bright natural killer cell	Innate
CYP27B1	CD56bright natural killer cell	Innate
EIF3M	CD56bright natural killer cell	Innate
CYP27A1	CD56dim natural killer cell	Innate
DDX55	CD56dim natural killer cell	Innate
DYRK2	CD56dim natural killer cell	Innate
RPL37A	CD56dim natural killer cell	Innate
NOTCH3	CD56dim natural killer cell	Innate
AKR7A3	CD56dim natural killer cell	Innate
GPRC5C	CD56dim natural killer cell	Innate
GRIN1	CD56dim natural killer cell	Innate
HLA-E	CD56dim natural killer cell	Innate
PORCN	CD56dim natural killer cell	Innate
PSMC4	CD56dim natural killer cell	Innate
UPP1	CD56dim natural killer cell	Innate
IL21R	CD56dim natural killer cell	Innate
KIR2DS1	CD56dim natural killer cell	Innate
KIR2DS2	CD56dim natural killer cell	Innate
KIR2DS5	CD56dim natural killer cell	Innate
GIPR	Eosinophil	Innate
KRT18P50	Eosinophil	Innate
LRMP	Eosinophil	Innate

FOSB	Eosinophil	Innate
RRP12	Eosinophil	Innate
GPR183	Eosinophil	Innate
NR4A3	Eosinophil	Innate
ST3GAL6	Eosinophil	Innate
DEPDC5	Eosinophil	Innate
PDE6C	Eosinophil	Innate
PKD2L2	Eosinophil	Innate
GPR65	Eosinophil	Innate
IL5RA	Eosinophil	Innate
P2RY14	Eosinophil	Innate
DACH1	Eosinophil	Innate
DAPK2	Eosinophil	Innate
EMR3	Eosinophil	Innate
ACADM	Immature dendritic cell	Innate
AHCYL1	Immature dendritic cell	Innate
ALDH1A2	Immature dendritic cell	Innate
ALDH3A2	Immature dendritic cell	Innate
ALDH9A1	Immature dendritic cell	Innate
ALOX15	Immature dendritic cell	Innate
AMT	Immature dendritic cell	Innate
ARL1	Immature dendritic cell	Innate
ATIC	Immature dendritic cell	Innate
ATP5A1	Immature dendritic cell	Innate
CAPZA1	Immature dendritic cell	Innate
LILRA5	Immature dendritic cell	Innate
RDX	Immature dendritic cell	Innate
RRAGD	Immature dendritic cell	Innate
TACSTD2	Immature dendritic cell	Innate
INPP5F	Immature dendritic cell	Innate
RAB38	Immature dendritic cell	Innate
PLAU	Immature dendritic cell	Innate
CSF3R	Immature dendritic cell	Innate
SLC18A2	Immature dendritic cell	Innate
AMPD2	Immature dendritic cell	Innate
CLTB	Immature dendritic cell	Innate
C1orf162	Immature dendritic cell	Innate
AIF1	Macrophage	Innate
CCL1	Macrophage	Innate
CCL14	Macrophage	Innate
CCL23	Macrophage	Innate
CCL26	Macrophage	Innate
CD300LB	Macrophage	Innate
CNR1	Macrophage	Innate

CNR2	Macrophage	Innate
EIF1	Macrophage	Innate
EIF4A1	Macrophage	Innate
FPR1	Macrophage	Innate
FPR2	Macrophage	Innate
FRAT2	Macrophage	Innate
GPR27	Macrophage	Innate
GPR77	Macrophage	Innate
RNASE2	Macrophage	Innate
MS4A2	Macrophage	Innate
BASP1	Macrophage	Innate
IGSF6	Macrophage	Innate
HK3	Macrophage	Innate
VNN1	Macrophage	Innate
FES	Macrophage	Innate
NPL	Macrophage	Innate
FZD2	Macrophage	Innate
FAM198B	Macrophage	Innate
HNMT	Macrophage	Innate
SLC15A3	Macrophage	Innate
CD4	Macrophage	Innate
TXNDC3	Macrophage	Innate
FRMD4A	Macrophage	Innate
CRYBB1	Macrophage	Innate
HRH1	Macrophage	Innate
WNT5B	Macrophage	Innate
ADAMTS3	Mast cell	Innate
CPA3	Mast cell	Innate
CMA1	Mast cell	Innate
CTSG	Mast cell	Innate
ARHGAP15	Mast cell	Innate
CPM	Mast cell	Innate
FCN1	Mast cell	Innate
FTL	Mast cell	Innate
HSPA6	Mast cell	Innate
ITGA9	Mast cell	Innate
RNASE3	Mast cell	Innate
S100A4	Mast cell	Innate
SIGLEC8	Mast cell	Innate
SLC6A4	Mast cell	Innate
PTGS2	Mast cell	Innate
EGR3	Mast cell	Innate
PILRA	Mast cell	Innate
CCR2	MDSC	Innate

CD14	MDSC	Innate
CD2	MDSC	Innate
CD86	MDSC	Innate
CXCR4	MDSC	Innate
FCGR2A	MDSC	Innate
FCGR2B	MDSC	Innate
FCGR3A	MDSC	Innate
FERMT3	MDSC	Innate
GPSM3	MDSC	Innate
IL18BP	MDSC	Innate
IL4R	MDSC	Innate
ITGAL	MDSC	Innate
ITGAM	MDSC	Innate
PARVG	MDSC	Innate
PSAP	MDSC	Innate
PTGER2	MDSC	Innate
PTGES2	MDSC	Innate
S100A8	MDSC	Innate
S100A9	MDSC	Innate
ASGR2	Monocyte	Innate
CFP	Monocyte	Innate
ASGR1	Monocyte	Innate
CD1D	Monocyte	Innate
UPK3A	Monocyte	Innate
ACTG1	Monocyte	Innate
ANXA5	Monocyte	Innate
ATP6V1B2	Monocyte	Innate
CFL1	Monocyte	Innate
DAZAP2	Monocyte	Innate
CTBS	Monocyte	Innate
EMR4P	Monocyte	Innate
HIVEP2	Monocyte	Innate
MARCKSL1	Monocyte	Innate
MBP	Monocyte	Innate
MMP15	Monocyte	Innate
PNPLA6	Monocyte	Innate
TMBIM6	Monocyte	Innate
PQBP1	Monocyte	Innate
TEX264	Monocyte	Innate
IKZF1	Monocyte	Innate
AKT3	Natural killer cell	Innate
AXL	Natural killer cell	Innate
BST2	Natural killer cell	Innate
CDH2	Natural killer cell	Innate

CRTAM	Natural killer cell	Innate
CSF2RA	Natural killer cell	Innate
CTSZ	Natural killer cell	Innate
CXCL1	Natural killer cell	Innate
CYTH1	Natural killer cell	Innate
DAXX	Natural killer cell	Innate
DGKH	Natural killer cell	Innate
DLL4	Natural killer cell	Innate
DPYD	Natural killer cell	Innate
ERBB3	Natural killer cell	Innate
F11R	Natural killer cell	Innate
FAM27A	Natural killer cell	Innate
FAM49A	Natural killer cell	Innate
FASLG	Natural killer cell	Innate
FCGR1A	Natural killer cell	Innate
FN1	Natural killer cell	Innate
FSTL1	Natural killer cell	Innate
FUCA1	Natural killer cell	Innate
GBP3	Natural killer cell	Innate
GLS2	Natural killer cell	Innate
GRB2	Natural killer cell	Innate
LST1	Natural killer cell	Innate
BCL2	Natural killer cell	Innate
CDC5L	Natural killer cell	Innate
FGF18	Natural killer cell	Innate
FUT5	Natural killer cell	Innate
FZR1	Natural killer cell	Innate
GAGE2	Natural killer cell	Innate
IGFBP5	Natural killer cell	Innate
KANK2	Natural killer cell	Innate
LDB3	Natural killer cell	Innate
BTN2A2	Natural killer T cell	Innate
CD101	Natural killer T cell	Innate
CD109	Natural killer T cell	Innate
CNPY3	Natural killer T cell	Innate
CNPY4	Natural killer T cell	Innate
CREB1	Natural killer T cell	Innate
CRTC2	Natural killer T cell	Innate
CRTC3	Natural killer T cell	Innate
CSF2	Natural killer T cell	Innate
KLRC1	Natural killer T cell	Innate
FUT4	Natural killer T cell	Innate
ICAM2	Natural killer T cell	Innate
IL32	Natural killer T cell	Innate

LAMP2	Natural killer T cell	Innate
LILRB5	Natural killer T cell	Innate
KLRG1	Natural killer T cell	Innate
HSPA4	Natural killer T cell	Innate
HSPB6	Natural killer T cell	Innate
ISM2	Natural killer T cell	Innate
ITIH2	Natural killer T cell	Innate
KDM4C	Natural killer T cell	Innate
KIR2DS4	Natural killer T cell	Innate
KIRREL3	Natural killer T cell	Innate
SDCBP	Natural killer T cell	Innate
NFATC2IP	Natural killer T cell	Innate
MICB	Natural killer T cell	Innate
KIR2DL1	Natural killer T cell	Innate
KIR2DL3	Natural killer T cell	Innate
KIR3DL1	Natural killer T cell	Innate
KIR3DL2	Natural killer T cell	Innate
NCR1	Natural killer T cell	Innate
FOSL1	Natural killer T cell	Innate
TSLP	Natural killer T cell	Innate
SLC7A7	Natural killer T cell	Innate
SPP1	Natural killer T cell	Innate
TREM2	Natural killer T cell	Innate
UBASH3A	Natural killer T cell	Innate
YBX2	Natural killer T cell	Innate
CCDC88A	Natural killer T cell	Innate
CLEC1A	Natural killer T cell	Innate
THBD	Natural killer T cell	Innate
PDPN	Natural killer T cell	Innate
VCAM1	Natural killer T cell	Innate
EMR1	Natural killer T cell	Innate
CREB5	Neutrophil	Innate
CDA	Neutrophil	Innate
CHST15	Neutrophil	Innate
S100A12	Neutrophil	Innate
APOBEC3A	Neutrophil	Innate
CASP5	Neutrophil	Innate
MMP25	Neutrophil	Innate
HAL	Neutrophil	Innate
C1orf183	Neutrophil	Innate
FFAR2	Neutrophil	Innate
MAK	Neutrophil	Innate
CXCR1	Neutrophil	Innate
STEAP4	Neutrophil	Innate

MGAM	Neutrophil	Innate
BTNL8	Neutrophil	Innate
CXCR2	Neutrophil	Innate
TNFRSF10C	Neutrophil	Innate
VNN3	Neutrophil	Innate
CBX6	Plasmacytoid dendritic cell	Innate
DAB2	Plasmacytoid dendritic cell	Innate
DDX17	Plasmacytoid dendritic cell	Innate
HIGD1A	Plasmacytoid dendritic cell	Innate
IDH3A	Plasmacytoid dendritic cell	Innate
IL3RA	Plasmacytoid dendritic cell	Innate
MAGED1	Plasmacytoid dendritic cell	Innate
NUCB2	Plasmacytoid dendritic cell	Innate
OFD1	Plasmacytoid dendritic cell	Innate
OGT	Plasmacytoid dendritic cell	Innate
PDIA4	Plasmacytoid dendritic cell	Innate
SERTAD2	Plasmacytoid dendritic cell	Innate
SIRPA	Plasmacytoid dendritic cell	Innate
TMED2	Plasmacytoid dendritic cell	Innate
ENG	Plasmacytoid dendritic cell	Innate
FCAR	Plasmacytoid dendritic cell	Innate
IGF1	Plasmacytoid dendritic cell	Innate
ITGA2B	Plasmacytoid dendritic cell	Innate
GABARAP	Plasmacytoid dendritic cell	Innate
GPX1	Plasmacytoid dendritic cell	Innate
KRT23	Plasmacytoid dendritic cell	Innate
PROK2	Plasmacytoid dendritic cell	Innate
RALB	Plasmacytoid dendritic cell	Innate
RETNLB	Plasmacytoid dendritic cell	Innate
RNF141	Plasmacytoid dendritic cell	Innate
SEC14L1	Plasmacytoid dendritic cell	Innate
SEPX1	Plasmacytoid dendritic cell	Innate
EMP3	Plasmacytoid dendritic cell	Innate
CD300LF	Plasmacytoid dendritic cell	Innate
ABTB1	Plasmacytoid dendritic cell	Innate
KLHL21	Plasmacytoid dendritic cell	Innate
PHRF1	Plasmacytoid dendritic cell	Innate

Table S4. 17 immune-related gene sets collected from the Immport.

Gene Symbol	Category
AZGP1	Antigen_Processing_and_Presentation
B2M	Antigen_Processing_and_Presentation
CALR	Antigen_Processing_and_Presentation
CANX	Antigen_Processing_and_Presentation
CD1A	Antigen_Processing_and_Presentation
CD1B	Antigen_Processing_and_Presentation
CD1C	Antigen_Processing_and_Presentation
CD1D	Antigen_Processing_and_Presentation
CD1E	Antigen_Processing_and_Presentation
CD4	Antigen_Processing_and_Presentation
CD8A	Antigen_Processing_and_Presentation
CD8B	Antigen_Processing_and_Presentation
CD74	Antigen_Processing_and_Presentation
CREB1	Antigen_Processing_and_Presentation
CTSB	Antigen_Processing_and_Presentation
CTSE	Antigen_Processing_and_Presentation
CTSL	Antigen_Processing_and_Presentation
CTSS	Antigen_Processing_and_Presentation
FCER1G	Antigen_Processing_and_Presentation
FCGRT	Antigen_Processing_and_Presentation
PDIA3	Antigen_Processing_and_Presentation
HFE	Antigen_Processing_and_Presentation
HLA-A	Antigen_Processing_and_Presentation
HLA-B	Antigen_Processing_and_Presentation
HLA-C	Antigen_Processing_and_Presentation
HLA-DMA	Antigen_Processing_and_Presentation
HLA-DMB	Antigen_Processing_and_Presentation
HLA-DOA	Antigen_Processing_and_Presentation
HLA-DOB	Antigen_Processing_and_Presentation
HLA-DPA1	Antigen_Processing_and_Presentation
HLA-DPB1	Antigen_Processing_and_Presentation
HLA-DQA1	Antigen_Processing_and_Presentation
HLA-DQA2	Antigen_Processing_and_Presentation
HLA-DQB1	Antigen_Processing_and_Presentation
HLA-DRA	Antigen_Processing_and_Presentation
HLA-DRB1	Antigen_Processing_and_Presentation
HLA-DRB3	Antigen_Processing_and_Presentation
HLA-DRB4	Antigen_Processing_and_Presentation
HLA-DRB5	Antigen_Processing_and_Presentation
HLA-E	Antigen_Processing_and_Presentation
HLA-F	Antigen_Processing_and_Presentation

HLA-G	Antigen_Processing_and_Presentation
HLA-H	Antigen_Processing_and_Presentation
MR1	Antigen_Processing_and_Presentation
HSPA1A	Antigen_Processing_and_Presentation
HSPA1B	Antigen_Processing_and_Presentation
HSPA1L	Antigen_Processing_and_Presentation
HSPA2	Antigen_Processing_and_Presentation
HSPA4	Antigen_Processing_and_Presentation
HSPA5	Antigen_Processing_and_Presentation
HSPA6	Antimicrobials
ORM1	Antimicrobials
TNF	Antimicrobials
CTSG	Antimicrobials
PRTN3	Antimicrobials
MAPK1	Antimicrobials
PML	Antimicrobials
AEN	Antimicrobials
CYBB	Antimicrobials
BPIFA2	Antimicrobials
ISG20	Antimicrobials
BCL3	Antimicrobials
ISG20L2	Antimicrobials
NOX5	Antimicrobials
NOX3	Antimicrobials
DUOX2	Antimicrobials
TLR3	Antimicrobials
TFRC	Antimicrobials
IFIH1	Antimicrobials
LRP1	Antimicrobials
TRIM5	Antimicrobials
IDO1	Antimicrobials
GDF15	Antimicrobials
NEDD4	Antimicrobials
ADIPOQ	Antimicrobials
STAT3	Antimicrobials
STAT1	Antimicrobials
IFNL2	Antimicrobials
SOCS3	Antimicrobials
SEMG1	Antimicrobials
TNFSF10	Antimicrobials
CCL20	Antimicrobials
SOCS1	Antimicrobials
RNASEL	Antimicrobials
IRF1	Antimicrobials

IL15	Antimicrobials
APOBEC3F	Antimicrobials
PLAAT4	Antimicrobials
CHIT1	Antimicrobials
IFNA1	Antimicrobials
CD40	Antimicrobials
TLR7	Antimicrobials
PPIA	Antimicrobials
HFE	Antimicrobials
ZYX	Antimicrobials
NLRX1	Antimicrobials
PGC	Antimicrobials
VEGFA	Antimicrobials
IKBKE	Antimicrobials
ISG15	Antimicrobials
DHX58	Antimicrobials
TNFAIP3	Antimicrobials
TFR2	Antimicrobials
FCN2	Antimicrobials
MUC4	Antimicrobials
F2R	Antimicrobials
ELN	Antimicrobials
IL27	Antimicrobials
MAPT	Antimicrobials
LYZ	Antimicrobials
CCL5	Antimicrobials
LEP	Antimicrobials
CYLD	Antimicrobials
KLKB1	Antimicrobials
CST4	Antimicrobials
CSRP1	Antimicrobials
MAPK14	Antimicrobials
JUN	Antimicrobials
ITGAV	Antimicrobials
IRF5	Antimicrobials
CCR6	Antimicrobials
IL12B	Antimicrobials
TLR8	Antimicrobials
GNLY	Antimicrobials
CD81	Antimicrobials
EIF2AK2	Antimicrobials
APOM	Antimicrobials
CACYBP	Antimicrobials
NOD1	Antimicrobials

MAPK8	Antimicrobials
MAPK3	Antimicrobials
BST2	Antimicrobials
BPHL	Antimicrobials
PLA2G2A	Antimicrobials
GRN	Antimicrobials
NEWENTRY	Antimicrobials
PDGFRA	Antimicrobials
GNAI1	Antimicrobials
WNT5A	Antimicrobials
FURIN	Antimicrobials
ADAR	Antimicrobials
TYK2	Antimicrobials
NOS2	Antimicrobials
TRAF3	Antimicrobials
TPT1	Antimicrobials
TPM2	Antimicrobials
NEO1	Antimicrobials
AHNAK	Antimicrobials
TLR1	Antimicrobials
TK2	Antimicrobials
PRDX2	Antimicrobials
MX2	Antimicrobials
FGF2	Antimicrobials
FGA	Antimicrobials
TCF7L2	Antimicrobials
F2RL1	Antimicrobials
TKFC	Antimicrobials
MSR1	Antimicrobials
NFKBIZ	Antimicrobials
LMBR1	Antimicrobials
EPPIN	Antimicrobials
SRC	Antimicrobials
MPO	Antimicrobials
ELAVL1	Antimicrobials
ROBO3	Antimicrobials
SP1	Antimicrobials
SOD1	Antimicrobials
PDF	Antimicrobials
DLL4	Antimicrobials
ECD	Antimicrobials
SLC11A1	Antimicrobials
DMBT1	Antimicrobials
STING1	Antimicrobials

SKIV2L	Antimicrobials
SEMG2	Antimicrobials
LTA	Antimicrobials
DES	Antimicrobials
DCK	Antimicrobials
DAXX	Antimicrobials
TNFRSF10A	Antimicrobials
TNFRSF10B	Antimicrobials
EED	Antimicrobials
CCL4	Antimicrobials
LIMS1	Antimicrobials
LALBA	Antimicrobials
APOBEC3H	Antimicrobials
TMPRSS6	Antimicrobials
SPINK5	Antimicrobials
MARCO	Antimicrobials
BECN1	Antimicrobials
TNFSF11	Antimicrobials
KNG1	Antimicrobials
CSK	Antimicrobials
KLRK1	Antimicrobials
KCNH2	Antimicrobials
JUND	Antimicrobials
JAK1	Antimicrobials
CREB1	Antimicrobials
CLDN4	Antimicrobials
CCL28	Antimicrobials
RNASE3	Antimicrobials
RN7SL1	Antimicrobials
IRF7	Antimicrobials
IREB2	Antimicrobials
ILK	Antimicrobials
IL18	Antimicrobials
IL17A	Antimicrobials
LTB4R	Antimicrobials
APOBEC3A	Antimicrobials
MASP2	Antimicrobials
TRIM27	Antimicrobials
RELA	Antimicrobials
IL7R	Antimicrobials
IL1A	Antimicrobials
PTX3	Antimicrobials
IFNAR2	Antimicrobials
IFN1@	Antimicrobials

SYTL1	Antimicrobials
APOBEC3C	Antimicrobials
DDX17	Antimicrobials
PTGS2	Antimicrobials
HTR1A	Antimicrobials
SEPTIN7	Antimicrobials
CD40LG	Antimicrobials
CD14	Antimicrobials
CD8A	Antimicrobials
CD4	Antimicrobials
MASP1	Antimicrobials
PROC	Antimicrobials
MAP2K2	Antimicrobials
MAP2K1	Antimicrobials
HRG	Antimicrobials
NDRG1	Antimicrobials
IRF9	Antimicrobials
TRIM22	Antimicrobials
LANCL1	Antimicrobials
PPP4C	Antimicrobials
HMOX1	Antimicrobials
HMGB1	Antimicrobials
HLA-B	Antimicrobials
RNASE7	Antimicrobials
ABCC4	Antimicrobials
HGF	Antimicrobials
HDAC1	Antimicrobials
IFNLR1	Antimicrobials
PLSCR1	Antimicrobials
B2M	Antimicrobials
BACH2	Antimicrobials
TANK	Antimicrobials
PIK3CG	Antimicrobials
ARRB1	Antimicrobials
RSAD2	Antimicrobials
STAB2	Antimicrobials
TBK1	Antimicrobials
PDYN	Antimicrobials
PDGFRB	Antimicrobials
PDCD1	Antimicrobials
PCSK2	Antimicrobials
PCSK1	Antimicrobials
ARG2	Antimicrobials
AQP9	Antimicrobials

FASLG	Antimicrobials
APOH	Antimicrobials
BIRC5	Antimicrobials
ANXA6	Antimicrobials
IL22	Antimicrobials
VTN	Antimicrobials
VIM	Antimicrobials
VCAM1	Antimicrobials
PRDX1	Antimicrobials
GFAP	Antimicrobials
GBP2	Antimicrobials
ALB	Antimicrobials
SLC29A3	Antimicrobials
OAS1	Antimicrobials
AGER	Antimicrobials
UNC93B1	Antimicrobials
TNFSF4	Antimicrobials
NOS1	Antimicrobials
ACTG1	Antimicrobials
ACTA1	Antimicrobials
ACO1	Antimicrobials
SERPINA3	Antimicrobials
CXCR1	Antimicrobials
CCL15	Antimicrobials
CCL14	Antimicrobials
CCL4	Antimicrobials
CCL16	Antimicrobials
CCL19	Antimicrobials
CCL13	Antimicrobials
CCL18	Antimicrobials
CCL17	Antimicrobials
CCL26	Antimicrobials
CCL22	Antimicrobials
CCR3	Antimicrobials
CCL28	Antimicrobials
CCL4L1	Antimicrobials
ACKR2	Antimicrobials
CCR7	Antimicrobials
CCL27	Antimicrobials
CCR8	Antimicrobials
ACKR4	Antimicrobials
CCR10	Antimicrobials
CCL2	Antimicrobials
CCL21	Antimicrobials

CCL7	Antimicrobials
CCL5	Antimicrobials
CCL3	Antimicrobials
CCL20	Antimicrobials
CCL11	Antimicrobials
CCR5	Antimicrobials
CCL23	Antimicrobials
CCL25	Antimicrobials
CCL1	Antimicrobials
CCL3L3	Antimicrobials
CCL4L2	Antimicrobials
CXCL12	Antimicrobials
XCL1	Antimicrobials
CCL8	Antimicrobials
CCL3L1	Antimicrobials
CCR1	Antimicrobials
CCL24	Antimicrobials
XCL2	Antimicrobials
CXCL1	Antimicrobials
CXCL10	Antimicrobials
CXCR4	Antimicrobials
CXCL2	Antimicrobials
CXCR6	Antimicrobials
CCR4	Antimicrobials
CXCL11	Antimicrobials
TAFA5	Antimicrobials
TAFA3	Antimicrobials
TAFA4	Antimicrobials
TAFA1	Antimicrobials
TAFA2	Antimicrobials
CCL15-CCL14	Antimicrobials
IL6	Antimicrobials
TNF	Antimicrobials
IL1B	Antimicrobials
IL18	Antimicrobials
PTK2B	Antimicrobials
VEGFA	Antimicrobials
IL4	Antimicrobials
CDH1	Antimicrobials
CD40	Antimicrobials
DEFB103B	Antimicrobials
F2RL1	Antimicrobials
MMP9	Antimicrobials
LTBP1	Antimicrobials

DEFB4A	Antimicrobials
TNFSF10	Antimicrobials
IL13	Antimicrobials
IL10	Antimicrobials
IL2	Antimicrobials
PPARG	Antimicrobials
FGR	Antimicrobials
MIF	Antimicrobials
CRP	Antimicrobials
JAK2	Antimicrobials
IL1A	Antimicrobials
PTK2	Antimicrobials
PTGDR	Antimicrobials
CD86	Antimicrobials
HCK	Antimicrobials
ARRB1	Antimicrobials
GNAI1	Antimicrobials
VDR	Antimicrobials
OLR1	Antimicrobials
GRK2	Antimicrobials
TXK	Antimicrobials
RNASE2	Antimicrobials
CD79A	BCRSignalingPathway
CD79B	BCRSignalingPathway
LYN	BCRSignalingPathway
SYK	BCRSignalingPathway
BTK	BCRSignalingPathway
BLNK	BCRSignalingPathway
VAV3	BCRSignalingPathway
VAV1	BCRSignalingPathway
VAV2	BCRSignalingPathway
RAC1	BCRSignalingPathway
RAC2	BCRSignalingPathway
RAC3	BCRSignalingPathway
PPP3CA	BCRSignalingPathway
PPP3CB	BCRSignalingPathway
PPP3CC	BCRSignalingPathway
CHP1	BCRSignalingPathway
PPP3R1	BCRSignalingPathway
PPP3R2	BCRSignalingPathway
CHP2	BCRSignalingPathway
NFAT5	BCRSignalingPathway
NFATC1	BCRSignalingPathway
NFATC2	BCRSignalingPathway

NFATC3	BCRSignalingPathway
NFATC4	BCRSignalingPathway
HRAS	BCRSignalingPathway
KRAS	BCRSignalingPathway
IGHD3-16	BCRSignalingPathway
IGHD3-22	BCRSignalingPathway
IGHD3-3	BCRSignalingPathway
IGHD3-9	BCRSignalingPathway
IGHD4-11	BCRSignalingPathway
IGHD4-17	BCRSignalingPathway
IGHD4-23	BCRSignalingPathway
IGHD4-4	BCRSignalingPathway
IGHD5-12	BCRSignalingPathway
IGHD5-18	BCRSignalingPathway
IGHD5-24	BCRSignalingPathway
IGHD5-5	BCRSignalingPathway
IGHD6-13	BCRSignalingPathway
IGHD6-19	BCRSignalingPathway
IGHD6-25	BCRSignalingPathway
IGHD6-6	BCRSignalingPathway
IGHD7-27	BCRSignalingPathway
IGHE	BCRSignalingPathway
IGHG1	BCRSignalingPathway
IGHG2	BCRSignalingPathway
IGHG3	BCRSignalingPathway
IGHG4	BCRSignalingPathway
IGHJ1	BCRSignalingPathway
IGHJ2	BCRSignalingPathway
IGHJ3	BCRSignalingPathway
IGHJ4	BCRSignalingPathway
IGHJ5	BCRSignalingPathway
IGHJ6	BCRSignalingPathway
IGHM	BCRSignalingPathway
IGH	BCRSignalingPathway
IGHV1-18	BCRSignalingPathway
IGHV1-2	BCRSignalingPathway
IGHV1-24	BCRSignalingPathway
IGHV1-3	BCRSignalingPathway
IGHV1-45	BCRSignalingPathway
IGHV1-46	BCRSignalingPathway
IGHV1-58	BCRSignalingPathway
IGHV1-69	BCRSignalingPathway
IGHV1-8	BCRSignalingPathway
IGHV1-38-4	BCRSignalingPathway

IGHV1-69-2	BCRSignalingPathway
IGHV2-26	BCRSignalingPathway
IGHV2-5	BCRSignalingPathway
IGHV2-70	BCRSignalingPathway
IGHV3-11	BCRSignalingPathway
IGHV3-13	BCRSignalingPathway
IGHV3-15	BCRSignalingPathway
IGHV3-16	BCRSignalingPathway
IGHV3-20	BCRSignalingPathway
IGHV3-21	BCRSignalingPathway
IGHV3-23	BCRSignalingPathway
IGHV3-30	BCRSignalingPathway
IGHV3-30-3	BCRSignalingPathway
IGHV3-30-5	BCRSignalingPathway
IGHV3-33	BCRSignalingPathway
IGHV3-35	BCRSignalingPathway
IGHV3-38	BCRSignalingPathway
IGHV3-43	BCRSignalingPathway
IGHV3-48	BCRSignalingPathway
IGHV3-49	BCRSignalingPathway
IGHV3-53	BCRSignalingPathway
IGHV3-64	BCRSignalingPathway
IGHV3-66	BCRSignalingPathway
IGHV3-7	BCRSignalingPathway
IGHV3-72	BCRSignalingPathway
IGHV3-73	BCRSignalingPathway
IGHV3-74	BCRSignalingPathway
IGHV3-9	BCRSignalingPathway
IGHV3-38-3	BCRSignalingPathway
IGHV3-69-1	BCRSignalingPathway
IGHV4-28	BCRSignalingPathway
IGHV4-30-1	BCRSignalingPathway
IGHV4-30-2	BCRSignalingPathway
IGHV4-30-4	BCRSignalingPathway
IGHV4-31	BCRSignalingPathway
IGHV4-34	BCRSignalingPathway
IGHV4-39	BCRSignalingPathway
IGHV4-4	BCRSignalingPathway
IGHV4-59	BCRSignalingPathway
IGHV4-61	BCRSignalingPathway
IGHV4-38-2	BCRSignalingPathway
IGHV5-51	BCRSignalingPathway
IGHV5-10-1	BCRSignalingPathway
IGHV6-1	BCRSignalingPathway

IGHV7-4-1	BCRSignalingPathway
IGHV7-81	BCRSignalingPathway
IGK	BCRSignalingPathway
IGKC	BCRSignalingPathway
IGKDEL	BCRSignalingPathway
IGKJ	BCRSignalingPathway
IGKJ1	BCRSignalingPathway
IGKJ2	BCRSignalingPathway
IGKJ3	BCRSignalingPathway
IGKJ4	BCRSignalingPathway
IGKJ5	BCRSignalingPathway
IGKV@	BCRSignalingPathway
IGKV1-12	BCRSignalingPathway
IGKV1-13	BCRSignalingPathway
IGKV1-16	BCRSignalingPathway
IGKV1-17	BCRSignalingPathway
IGKV1-27	BCRSignalingPathway
IGKV1-33	BCRSignalingPathway
IGKV1-37	BCRSignalingPathway
IGKV1-39	BCRSignalingPathway
IGKV1-5	BCRSignalingPathway
IGKV1-6	BCRSignalingPathway
IGKV1-8	BCRSignalingPathway
IGKV1-9	BCRSignalingPathway
IGKV1D-12	BCRSignalingPathway
IGKV1D-13	BCRSignalingPathway
IGKV1D-16	BCRSignalingPathway
IGKV1D-17	BCRSignalingPathway
IGKV1D-33	BCRSignalingPathway
IGKV1D-37	BCRSignalingPathway
IGKV1D-39	BCRSignalingPathway
IGKV1D-42	BCRSignalingPathway
IGKV1D-43	BCRSignalingPathway
IGKV1D-8	BCRSignalingPathway
IGKV2-24	BCRSignalingPathway
IGKV2-28	BCRSignalingPathway
IGKV2-30	BCRSignalingPathway
IGKV2-40	BCRSignalingPathway
IGKV2D-24	BCRSignalingPathway
IGKV2D-28	BCRSignalingPathway
IGKV2D-29	BCRSignalingPathway
IGKV2D-30	BCRSignalingPathway
IGKV2D-40	BCRSignalingPathway
IGKV3-11	BCRSignalingPathway

IGKV3-15	BCRSignalingPathway
IGKV3-20	BCRSignalingPathway
IGKV3-7	BCRSignalingPathway
IGKV3D-11	BCRSignalingPathway
IGKV3D-15	BCRSignalingPathway
IGKV3D-20	BCRSignalingPathway
IGKV3D-7	BCRSignalingPathway
IGKV4-1	BCRSignalingPathway
IGKV5-2	BCRSignalingPathway
IGKV6-21	BCRSignalingPathway
IGKV6D-21	BCRSignalingPathway
IGKV6D-41	BCRSignalingPathway
IGL	BCRSignalingPathway
IGLC1	BCRSignalingPathway
IGLC2	BCRSignalingPathway
IGLC3	BCRSignalingPathway
IGLC6	BCRSignalingPathway
IGLC7	BCRSignalingPathway
IGLJ	BCRSignalingPathway
IGLJ1	BCRSignalingPathway
IGLJ2	BCRSignalingPathway
IGLJ3	BCRSignalingPathway
IGLJ4	BCRSignalingPathway
IGLJ5	BCRSignalingPathway
IGLJ6	BCRSignalingPathway
IGLJ7	BCRSignalingPathway
IGLV@	BCRSignalingPathway
IGLV1-36	BCRSignalingPathway
IGLV1-40	BCRSignalingPathway
IGLV1-44	BCRSignalingPathway
IGLV1-47	BCRSignalingPathway
IGLV1-50	BCRSignalingPathway
IGLV1-51	BCRSignalingPathway
IGLV10-54	BCRSignalingPathway
IGLV11-55	BCRSignalingPathway
IGLV2-11	BCRSignalingPathway
IGLV2-14	BCRSignalingPathway
IGLV2-18	BCRSignalingPathway
IGLV2-23	BCRSignalingPathway
IGLV2-33	BCRSignalingPathway
IGLV2-8	BCRSignalingPathway
IGLV3-1	BCRSignalingPathway
IGLV3-10	BCRSignalingPathway
IGLV3-12	BCRSignalingPathway

IGLV3-16	BCRSignalingPathway
IGLV3-19	BCRSignalingPathway
IGLV3-21	BCRSignalingPathway
IGLV3-22	BCRSignalingPathway
IGLV3-25	BCRSignalingPathway
IGLV3-27	BCRSignalingPathway
IGLV3-32	BCRSignalingPathway
IGLV3-9	BCRSignalingPathway
IGLV4-3	BCRSignalingPathway
IGLV4-60	BCRSignalingPathway
IGLV4-69	BCRSignalingPathway
IGLV5-37	BCRSignalingPathway
IGLV5-39	BCRSignalingPathway
IGLV5-45	BCRSignalingPathway
IGLV5-48	BCRSignalingPathway
IGLV5-52	BCRSignalingPathway
IGLV6-57	BCRSignalingPathway
IGLV7-43	BCRSignalingPathway
IGLV7-46	BCRSignalingPathway
IGLV8-61	BCRSignalingPathway
IGLV9-49	BCRSignalingPathway
C3	Chemokines
C5	Chemokines
CAMP	Chemokines
CCL1	Chemokines
CCL11	Chemokines
CCL13	Chemokines
CCL14	Chemokines
CCL15-CCL14	Chemokines
CCL15	Chemokines
CCL16	Chemokines
CCL17	Chemokines
CCL18	Chemokines
CCL19	Chemokines
CCL2	Chemokines
CCL20	Chemokines
CCL21	Chemokines
CCL22	Chemokines
CCL23	Chemokines
CCL24	Chemokines
CCL25	Chemokines
CCL26	Chemokines
CCL27	Chemokines
CCL28	Chemokines

CCL3	Chemokines
CCL3L1	Chemokines
CCL3P1	Chemokines
CCL3L3	Chemokines
CCL4	Chemokines
CCL4L2	Chemokines
CCL4L1	Chemokines
CCL5	Chemokines
CCL7	Chemokines
CCL8	Chemokines
CKLF	Chemokines
CMA1	Chemokines
CTSG	Chemokines
CX3CL1	Chemokines
CXCL1	Chemokines
CXCL10	Chemokines
CXCL11	Chemokines
CXCL12	Chemokines
CXCL13	Chemokines
CXCL14	Chemokines
CXCL16	Chemokines
CXCL17	Chemokines
CXCL2	Chemokines
CXCL3	Chemokines
CXCL5	Chemokines
CXCL6	Chemokines
CXCL9	Chemokines
CCN1	Chemokines
DEFA1	Chemokines
DEFA3	Chemokines
DEFA5	Chemokines
DEFB1	Chemokines
DEFB103B	Chemokines
DEFB104A	Chemokines
DEFB4A	Chemokines
EDN1	Chemokines
EDN2	Chemokines
EDN3	Chemokines
FGF10	Chemokines
FGF2	Chemokines
HTN3	Chemokines
CXCL8	Chemokines
LECT2	Chemokines
PF4	Chemokines

PF4V1	Chemokines
PLAU	Chemokines
PPBP	Chemokines
PPBPP1	Chemokines
PROK2	Chemokines
RNASE2	Chemokines
SAA1	Chemokines
SAA2	Chemokines
SBDS	Chemokines
SEMA3A	Chemokines
SEMA3B	Chemokines
SEMA3C	Chemokines
SEMA3D	Chemokines
SEMA3E	Chemokines
SEMA3F	Chemokines
SEMA3G	Chemokines
SEMA4A	Chemokines
SEMA4B	Chemokines
SEMA4C	Chemokines
SEMA4D	Chemokines
SEMA4F	Chemokines
SEMA4G	Chemokines
SEMA5A	Chemokines
SEMA5B	Chemokines
SEMA6A	Chemokines
SEMA6B	Chemokines
SEMA6C	Chemokines
SEMA6D	Chemokines
SEMA7A	Chemokines
SLIT1	Chemokines
SLIT2	Chemokines
TNC	Chemokines
TYMP	Chemokines
XCL1	Chemokines
XCL2	Chemokines
C5AR1	Chemokine_Receptors
ACKR2	Chemokine_Receptors
CCR1	Chemokine_Receptors
CCR10	Chemokine_Receptors
CCR3	Chemokine_Receptors
CCR4	Chemokine_Receptors
CCR5	Chemokine_Receptors
CCR6	Chemokine_Receptors
CCR7	Chemokine_Receptors

CCR8	Chemokine_Receptors
CCR9	Chemokine_Receptors
ACKR4	Chemokine_Receptors
CCRL2	Chemokine_Receptors
CMKLR1	Chemokine_Receptors
CX3CR1	Chemokine_Receptors
CXCR3	Chemokine_Receptors
CXCR4	Chemokine_Receptors
CXCR5	Chemokine_Receptors
CXCR6	Chemokine_Receptors
ACKR3	Chemokine_Receptors
CYSLTR1	Chemokine_Receptors
CYSLTR2	Chemokine_Receptors
ACKR1	Chemokine_Receptors
EDNRA	Chemokine_Receptors
EDNRB	Chemokine_Receptors
FPR1	Chemokine_Receptors
FPR2	Chemokine_Receptors
FPR2	Chemokine_Receptors
GPR17	Chemokine_Receptors
GPR32	Chemokine_Receptors
GPR33	Chemokine_Receptors
PTGDR2	Chemokine_Receptors
C5AR2	Chemokine_Receptors
CXCR1	Chemokine_Receptors
CXCR2	Chemokine_Receptors
LTB4R	Chemokine_Receptors
LTB4R2	Chemokine_Receptors
PLAUR	Chemokine_Receptors
PLXNA1	Chemokine_Receptors
PLXNA2	Chemokine_Receptors
PLXNA3	Chemokine_Receptors
PLXNA4	Chemokine_Receptors
PLXNB1	Chemokine_Receptors
PLXNB2	Chemokine_Receptors
PLXNB3	Chemokine_Receptors
PLXNC1	Chemokine_Receptors
PLXND1	Chemokine_Receptors
PTAFR	Chemokine_Receptors
ROBO1	Chemokine_Receptors
ROBO2	Chemokine_Receptors
ROBO3	Chemokine_Receptors
RXFP3	Chemokine_Receptors
XCR1	Chemokine_Receptors

ADIPOQ	Cytokines
ADM	Cytokines
ADM2	Cytokines
AGRP	Cytokines
AGT	Cytokines
AMBN	Cytokines
AMELX	Cytokines
AMH	Cytokines
ANGPTL5	Cytokines
ANGPTL7	Cytokines
APLN	Cytokines
AREG	Cytokines
MANF	Cytokines
CDNF	Cytokines
ARTN	Cytokines
AVP	Cytokines
AZU1	Cytokines
BDNF	Cytokines
BMP1	Cytokines
BMP10	Cytokines
BMP15	Cytokines
BMP2	Cytokines
BMP3	Cytokines
BMP4	Cytokines
BMP5	Cytokines
BMP6	Cytokines
BMP7	Cytokines
BMP8A	Cytokines
BMP8B	Cytokines
BTC	Cytokines
MYDGF	Cytokines
C3	Cytokines
C5	Cytokines
CALCA	Cytokines
CALCB	Cytokines
CAMP	Cytokines
CAT	Cytokines
CCK	Cytokines
CCL1	Cytokines
CCL11	Cytokines
CCL13	Cytokines
CCL14	Cytokines
CCL15-CCL14	Cytokines
CCL15	Cytokines

CCL16	Cytokines
CCL17	Cytokines
CCL18	Cytokines
CCL19	Cytokines
CCL2	Cytokines
CCL20	Cytokines
CCL21	Cytokines
CCL22	Cytokines
CCL23	Cytokines
CCL24	Cytokines
CCL25	Cytokines
CCL26	Cytokines
CCL27	Cytokines
CCL28	Cytokines
CCL3	Cytokines
CCL3L1	Cytokines
CCL3P1	Cytokines
CCL3L3	Cytokines
CCL4	Cytokines
CCL4L2	Cytokines
CCL4L1	Cytokines
CCL5	Cytokines
CCL7	Cytokines
CCL8	Cytokines
CD320	Cytokines
CD40LG	Cytokines
CD70	Cytokines
ADA2	Cytokines
CER1	Cytokines
CGA	Cytokines
CGB3	Cytokines
CGB1	Cytokines
CGB2	Cytokines
CGB5	Cytokines
CGB7	Cytokines
CGB8	Cytokines
CHGA	Cytokines
CHGB	Cytokines
CKLF	Cytokines
CLCF1	Cytokines
CLEC11A	Cytokines
CMA1	Cytokines
CMTM1	Cytokines
CMTM2	Cytokines

CMTM3	Cytokines
CMTM4	Cytokines
CMTM5	Cytokines
CMTM6	Cytokines
CMTM7	Cytokines
CMTM8	Cytokines
CNTF	Cytokines
CORT	Cytokines
CRH	Cytokines
CSF1	Cytokines
CSF2	Cytokines
CSF3	Cytokines
CSH1	Cytokines
CSH2	Cytokines
CSHL1	Cytokines
CSPG5	Cytokines
CTF1	Cytokines
CCN2	Cytokines
CTSG	Cytokines
CX3CL1	Cytokines
CXCL1	Cytokines
CXCL10	Cytokines
CXCL11	Cytokines
CXCL12	Cytokines
CXCL13	Cytokines
CXCL14	Cytokines
CXCL16	Cytokines
CXCL17	Cytokines
CXCL2	Cytokines
CXCL3	Cytokines
CXCL5	Cytokines
CXCL6	Cytokines
CXCL9	Cytokines
CCN1	Cytokines
DEFA1	Cytokines
DEFA3	Cytokines
DEFA5	Cytokines
DEFB1	Cytokines
DEFB103B	Cytokines
DEFB104A	Cytokines
DEFB4A	Cytokines
DKK1	Cytokines
EBI3	Cytokines
EDN1	Cytokines

EDN2	Cytokines
EDN3	Cytokines
EGF	Cytokines
EPGN	Cytokines
EPO	Cytokines
EREG	Cytokines
ESM1	Cytokines
FAM3B	Cytokines
FAM3C	Cytokines
FAM3D	Cytokines
FASLG	Cytokines
FGF1	Cytokines
FGF10	Cytokines
FGF11	Cytokines
FGF12	Cytokines
FGF13	Cytokines
FGF14	Cytokines
FGF16	Cytokines
FGF17	Cytokines
FGF18	Cytokines
FGF19	Cytokines
FGF2	Cytokines
FGF20	Cytokines
FGF21	Cytokines
FGF22	Cytokines
FGF23	Cytokines
FGF3	Cytokines
FGF4	Cytokines
FGF5	Cytokines
FGF6	Cytokines
FGF7	Cytokines
FGF8	Cytokines
FGF9	Cytokines
VEGFD	Cytokines
FIGNL2	Cytokines
FLT3LG	Cytokines
FSHB	Cytokines
GAL	Cytokines
GALP	Cytokines
GAST	Cytokines
GCG	Cytokines
GDF1	Cytokines
GDF10	Cytokines
GDF11	Cytokines

GDF15	Cytokines
GDF2	Cytokines
GDF3	Cytokines
GDF5	Cytokines
GDF6	Cytokines
GDF7	Cytokines
GDF9	Cytokines
GDNF	Cytokines
GH1	Cytokines
GH2	Cytokines
GHRH	Cytokines
GHRL	Cytokines
GIP	Cytokines
GKN1	Cytokines
GMFB	Cytokines
GMFG	Cytokines
GNRH1	Cytokines
GNRH2	Cytokines
GPHA2	Cytokines
GPHB5	Cytokines
GPI	Cytokines
GREM1	Cytokines
GREM2	Cytokines
GRN	Cytokines
GRP	Cytokines
GUCA2A	Cytokines
HAMP	Cytokines
HBEGF	Cytokines
HDGF	Cytokines
HDGFL3	Cytokines
HGF	Cytokines
HTN3	Cytokines
IAPP	Cytokines
IFNA1	Cytokines
IFNA10	Cytokines
IFNA13	Cytokines
IFNA14	Cytokines
IFNA16	Cytokines
IFNA17	Cytokines
IFNA2	Cytokines
IFNA21	Cytokines
IFNA4	Cytokines
IFNA5	Cytokines
IFNA6	Cytokines

IFNA7	Cytokines
IFNA8	Cytokines
IFNB1	Cytokines
IFNE	Cytokines
IFNG	Cytokines
IFNK	Cytokines
IFNW1	Cytokines
IGF1	Cytokines
IGF2	Cytokines
IL10	Cytokines
IL11	Cytokines
IL12A	Cytokines
IL12B	Cytokines
IL13	Cytokines
IL15	Cytokines
IL16	Cytokines
IL17A	Cytokines
IL17B	Cytokines
IL17C	Cytokines
IL17D	Cytokines
IL17F	Cytokines
IL18	Cytokines
IL19	Cytokines
IL1A	Cytokines
IL1B	Cytokines
IL1F10	Cytokines
IL36RN	Cytokines
IL36A	Cytokines
IL37	Cytokines
IL36B	Cytokines
IL36G	Cytokines
IL1RN	Cytokines
IL2	Cytokines
IL20	Cytokines
IL21	Cytokines
IL22	Cytokines
IL23A	Cytokines
IL24	Cytokines
IL25	Cytokines
IL26	Cytokines
IL27	Cytokines
IFNL2	Cytokines
IFNL3	Cytokines
IFNL1	Cytokines

IL3	Cytokines
IL31	Cytokines
IL32	Cytokines
IL33	Cytokines
IL34	Cytokines
IL4	Cytokines
IL5	Cytokines
IL6	Cytokines
IL6ST	Cytokines
IL7	Cytokines
CXCL8	Cytokines
IL9	Cytokines
INHA	Cytokines
INHBA	Cytokines
INHBB	Cytokines
INHBC	Cytokines
INHBE	Cytokines
INS	Cytokines
INS-IGF2	Cytokines
INSL3	Cytokines
INSL4	Cytokines
INSL5	Cytokines
INSL6	Cytokines
JAG1	Cytokines
JAG2	Cytokines
FGF7P6	Cytokines
FGF7P3	Cytokines
KITLG	Cytokines
KL	Cytokines
LACRT	Cytokines
LECT2	Cytokines
LEFTY1	Cytokines
LEFTY2	Cytokines
LEP	Cytokines
LHB	Cytokines
LIF	Cytokines
LRSAM1	Cytokines
LTA	Cytokines
LTB	Cytokines
LTBP1	Cytokines
LTBP2	Cytokines
LTBP3	Cytokines
LTBP4	Cytokines
MDK	Cytokines

MIA	Cytokines
MIF	Cytokines
MLN	Cytokines
MSTN	Cytokines
NAMPT	Cytokines
NDP	Cytokines
NENF	Cytokines
NGF	Cytokines
NMB	Cytokines
NODAL	Cytokines
CCN3	Cytokines
NPFF	Cytokines
NPPA	Cytokines
NPPB	Cytokines
NPPC	Cytokines
NPY	Cytokines
NRG1	Cytokines
NRG2	Cytokines
NRG3	Cytokines
NRG4	Cytokines
NRTN	Cytokines
NTF3	Cytokines
NTF4	Cytokines
NTS	Cytokines
NUDT6	Cytokines
OGN	Cytokines
OSGIN1	Cytokines
OSM	Cytokines
OSTN	Cytokines
OXT	Cytokines
ENDOU	Cytokines
PDGFA	Cytokines
PDGFB	Cytokines
PDGFC	Cytokines
PDGFD	Cytokines
PDGFRA	Cytokines
PDGFRB	Cytokines
PDGFRL	Cytokines
PDYN	Cytokines
PENK	Cytokines
PF4	Cytokines
PF4V1	Cytokines
PGF	Cytokines
PLAU	Cytokines

PMCH	Cytokines
PNOC	Cytokines
POMC	Cytokines
PPBP	Cytokines
PPBPP1	Cytokines
PPBPP2	Cytokines
PPY	Cytokines
PRL	Cytokines
PRLH	Cytokines
PROK1	Cytokines
PROK2	Cytokines
PSPN	Cytokines
PTH	Cytokines
PTH2	Cytokines
PTHLH	Cytokines
PTN	Cytokines
PYY	Cytokines
QRFP	Cytokines
RABEP1	Cytokines
RABEP2	Cytokines
REG1A	Cytokines
RETN	Cytokines
RETNLB	Cytokines
RLN1	Cytokines
RLN2	Cytokines
RLN3	Cytokines
RNASE2	Cytokines
S100A6	Cytokines
SAA1	Cytokines
SAA2	Cytokines
SBDS	Cytokines
SCG2	Cytokines
SCGB3A1	Cytokines
SCT	Cytokines
AIMP1	Cytokines
SECTM1	Cytokines
SEMA3A	Cytokines
SEMA3B	Cytokines
SEMA3C	Cytokines
SEMA3D	Cytokines
SEMA3E	Cytokines
SEMA3F	Cytokines
SEMA3G	Cytokines
SEMA4A	Cytokines

SEMA4B	Cytokines
SEMA4C	Cytokines
SEMA4D	Cytokines
SEMA4F	Cytokines
SEMA4G	Cytokines
SEMA5A	Cytokines
SEMA5B	Cytokines
SEMA6A	Cytokines
SEMA6B	Cytokines
SEMA6C	Cytokines
SEMA6D	Cytokines
SEMA7A	Cytokines
SLIT1	Cytokines
SLIT2	Cytokines
SLURP1	Cytokines
SPP1	Cytokines
SST	Cytokines
STC1	Cytokines
STC2	Cytokines
TAC1	Cytokines
TDGF1	Cytokines
TDGF1P3	Cytokines
TG	Cytokines
TGFA	Cytokines
TGFB1	Cytokines
TGFB2	Cytokines
TGFB3	Cytokines
THPO	Cytokines
TNC	Cytokines
TNF	Cytokines
TNFRSF11B	Cytokines
TNFSF10	Cytokines
TNFSF11	Cytokines
TNFSF12	Cytokines
TNFSF13	Cytokines
TNFSF13B	Cytokines
TNFSF14	Cytokines
TNFSF15	Cytokines
TNFSF18	Cytokines
TNFSF4	Cytokines
TNFSF8	Cytokines
TNFSF9	Cytokines
TOR2A	Cytokines
TRH	Cytokines

TSHB	Cytokines
TSLP	Cytokines
TXLNA	Cytokines
TYMP	Cytokines
UCN	Cytokines
UCN2	Cytokines
UCN3	Cytokines
UTS2	Cytokines
UTS2B	Cytokines
VEGFA	Cytokines
VEGFB	Cytokines
VEGFC	Cytokines
VGF	Cytokines
VIP	Cytokines
XCL1	Cytokines
XCL2	Cytokines
ACVR1B	Cytokine_Receptors
ACVR1C	Cytokine_Receptors
ACVR2A	Cytokine_Receptors
ACVR2B	Cytokine_Receptors
ACVRL1	Cytokine_Receptors
ADCYAP1R1	Cytokine_Receptors
ADIPOR1	Cytokine_Receptors
ADIPOR2	Cytokine_Receptors
ADRB1	Cytokine_Receptors
ADRB2	Cytokine_Receptors
AGTR1	Cytokine_Receptors
AGTR2	Cytokine_Receptors
AMHR2	Cytokine_Receptors
ANGPT1	Cytokine_Receptors
ANGPT4	Cytokine_Receptors
ANGPTL1	Cytokine_Receptors
ANGPTL2	Cytokine_Receptors
ANGPTL3	Cytokine_Receptors
ANGPTL4	Cytokine_Receptors
ANGPTL6	Cytokine_Receptors
APLNR	Cytokine_Receptors
AR	Cytokine_Receptors
AVPR1A	Cytokine_Receptors
AVPR1B	Cytokine_Receptors
AVPR2	Cytokine_Receptors
BMPR1A	Cytokine_Receptors
BMPR1B	Cytokine_Receptors
BMPR2	Cytokine_Receptors

BRD8	Cytokine_Receptors
C3AR1	Cytokine_Receptors
C5AR1	Cytokine_Receptors
CALCR	Cytokine_Receptors
CALCRL	Cytokine_Receptors
ACKR2	Cytokine_Receptors
CCR1	Cytokine_Receptors
CCR10	Cytokine_Receptors
CCR3	Cytokine_Receptors
CCR4	Cytokine_Receptors
CCR5	Cytokine_Receptors
CCR6	Cytokine_Receptors
CCR7	Cytokine_Receptors
CCR8	Cytokine_Receptors
CCR9	Cytokine_Receptors
ACKR4	Cytokine_Receptors
CCRL2	Cytokine_Receptors
CD40	Cytokine_Receptors
CMKLR1	Cytokine_Receptors
CNTFR	Cytokine_Receptors
CRHR1	Cytokine_Receptors
CRHR2	Cytokine_Receptors
CRIM1	Cytokine_Receptors
CRLF1	Cytokine_Receptors
CRLF2	Cytokine_Receptors
CRLF3	Cytokine_Receptors
CSF1R	Cytokine_Receptors
CSF2RA	Cytokine_Receptors
CSF2RB	Cytokine_Receptors
CSF3R	Cytokine_Receptors
CX3CR1	Cytokine_Receptors
CXCR3	Cytokine_Receptors
CXCR4	Cytokine_Receptors
CXCR5	Cytokine_Receptors
CXCR6	Cytokine_Receptors
ACKR3	Cytokine_Receptors
CYSLTR1	Cytokine_Receptors
CYSLTR2	Cytokine_Receptors
ACKR1	Cytokine_Receptors
EDNRA	Cytokine_Receptors
EDNRB	Cytokine_Receptors
EGFR	Cytokine_Receptors
ENG	Cytokine_Receptors
EPOR	Cytokine_Receptors

ESR1	Cytokine_Receptors
ESR2	Cytokine_Receptors
ESRRA	Cytokine_Receptors
ESRRB	Cytokine_Receptors
ESRRG	Cytokine_Receptors
FGFR1	Cytokine_Receptors
FGFR2	Cytokine_Receptors
FGFR3	Cytokine_Receptors
FGFR4	Cytokine_Receptors
FGFRL1	Cytokine_Receptors
FLT1	Cytokine_Receptors
FLT3	Cytokine_Receptors
FLT4	Cytokine_Receptors
FPR1	Cytokine_Receptors
FPR2	Cytokine_Receptors
FPR2	Cytokine_Receptors
FSHR	Cytokine_Receptors
GALR2	Cytokine_Receptors
GALR3	Cytokine_Receptors
GCGR	Cytokine_Receptors
GHR	Cytokine_Receptors
GHRHR	Cytokine_Receptors
GHSR	Cytokine_Receptors
GIPR	Cytokine_Receptors
GLP1R	Cytokine_Receptors
GLP2R	Cytokine_Receptors
GNRHR	Cytokine_Receptors
GPER1	Cytokine_Receptors
GPR17	Cytokine_Receptors
GPR32	Cytokine_Receptors
GPR33	Cytokine_Receptors
PTGDR2	Cytokine_Receptors
C5AR2	Cytokine_Receptors
HNF4A	Cytokine_Receptors
HNF4G	Cytokine_Receptors
HTR3A	Cytokine_Receptors
HTR3B	Cytokine_Receptors
HTR3C	Cytokine_Receptors
HTR3D	Cytokine_Receptors
HTR3E	Cytokine_Receptors
IFNAR1	Cytokine_Receptors
IFNAR2	Cytokine_Receptors
IFNGR1	Cytokine_Receptors
IFNGR2	Cytokine_Receptors

IGF1R	Cytokine_Receptors
IGF2R	Cytokine_Receptors
IL10RA	Cytokine_Receptors
IL10RB	Cytokine_Receptors
IL11RA	Cytokine_Receptors
IL12RB1	Cytokine_Receptors
IL12RB2	Cytokine_Receptors
IL13RA1	Cytokine_Receptors
IL13RA2	Cytokine_Receptors
IL15RA	Cytokine_Receptors
IL2RB	Cytokine_Receptors
IL17RA	Cytokine_Receptors
IL17RB	Cytokine_Receptors
IL17RC	Cytokine_Receptors
IL17RD	Cytokine_Receptors
IL17RE	Cytokine_Receptors
IL18R1	Cytokine_Receptors
IL18RAP	Cytokine_Receptors
IL1R1	Cytokine_Receptors
IL1R2	Cytokine_Receptors
IL1RAP	Cytokine_Receptors
IL1RL1	Cytokine_Receptors
IL1RL2	Cytokine_Receptors
IL20RA	Cytokine_Receptors
IL20RB	Cytokine_Receptors
IL21R	Cytokine_Receptors
IL22RA1	Cytokine_Receptors
IL22RA2	Cytokine_Receptors
IL23R	Cytokine_Receptors
IL27RA	Cytokine_Receptors
IFNLR1	Cytokine_Receptors
IL2RA	Cytokine_Receptors
IL2RB	Cytokine_Receptors
IL2RG	Cytokine_Receptors
IL31RA	Cytokine_Receptors
IL3RA	Cytokine_Receptors
IL4R	Cytokine_Receptors
IL5RA	Cytokine_Receptors
IL6R	Cytokine_Receptors
IL7R	Cytokine_Receptors
CXCR1	Cytokine_Receptors
CXCR2	Cytokine_Receptors
IL9R	Cytokine_Receptors
INSR	Cytokine_Receptors

KDR	Cytokine_Receptors
LEPR	Cytokine_Receptors
LGR4	Cytokine_Receptors
LGR5	Cytokine_Receptors
LGR6	Cytokine_Receptors
LHCGR	Cytokine_Receptors
LIFR	Cytokine_Receptors
LTB4R	Cytokine_Receptors
LTB4R2	Cytokine_Receptors
LTBR	Cytokine_Receptors
MC1R	Cytokine_Receptors
MC2R	Cytokine_Receptors
MC3R	Cytokine_Receptors
MC4R	Cytokine_Receptors
MCHR1	Cytokine_Receptors
MCHR2	Cytokine_Receptors
MET	Cytokine_Receptors
MLNR	Cytokine_Receptors
MPL	Cytokine_Receptors
MTNR1A	Cytokine_Receptors
MTNR1B	Cytokine_Receptors
NGFR	Cytokine_Receptors
NMBR	Cytokine_Receptors
NPR1	Cytokine_Receptors
NPR3	Cytokine_Receptors
NR0B1	Cytokine_Receptors
NR0B2	Cytokine_Receptors
NR1D1	Cytokine_Receptors
NR1D2	Cytokine_Receptors
NR1H2	Cytokine_Receptors
NR1H3	Cytokine_Receptors
NR1H4	Cytokine_Receptors
NR1I2	Cytokine_Receptors
NR1I3	Cytokine_Receptors
NR2C1	Cytokine_Receptors
NR2C2	Cytokine_Receptors
NR2E1	Cytokine_Receptors
NR2E3	Cytokine_Receptors
NR2F1	Cytokine_Receptors
NR2F2	Cytokine_Receptors
NR2F6	Cytokine_Receptors
NR3C1	Cytokine_Receptors
NR3C2	Cytokine_Receptors
NR4A1	Cytokine_Receptors

NR4A2	Cytokine_Receptors
NR4A3	Cytokine_Receptors
NR5A1	Cytokine_Receptors
NR5A2	Cytokine_Receptors
NR6A1	Cytokine_Receptors
NRP1	Cytokine_Receptors
NRP2	Cytokine_Receptors
OGFR	Cytokine_Receptors
OPRD1	Cytokine_Receptors
OPRK1	Cytokine_Receptors
OPRL1	Cytokine_Receptors
OPRM1	Cytokine_Receptors
OSMR	Cytokine_Receptors
OXTR	Cytokine_Receptors
PGR	Cytokine_Receptors
PGRMC2	Cytokine_Receptors
PLAUR	Cytokine_Receptors
PLXNA1	Cytokine_Receptors
PLXNA2	Cytokine_Receptors
PLXNA3	Cytokine_Receptors
PLXNA4	Cytokine_Receptors
PLXNB1	Cytokine_Receptors
PLXNB2	Cytokine_Receptors
PLXNB3	Cytokine_Receptors
PLXNC1	Cytokine_Receptors
PLXND1	Cytokine_Receptors
PPARA	Cytokine_Receptors
PPARD	Cytokine_Receptors
PPARG	Cytokine_Receptors
PRLHR	Cytokine_Receptors
PRLR	Cytokine_Receptors
PTAFR	Cytokine_Receptors
PTGDR	Cytokine_Receptors
PTGDS	Cytokine_Receptors
PTGER1	Cytokine_Receptors
PTGER2	Cytokine_Receptors
PTGER3	Cytokine_Receptors
PTGER4	Cytokine_Receptors
PTGFR	Cytokine_Receptors
PTH1R	Cytokine_Receptors
PTH2R	Cytokine_Receptors
RARA	Cytokine_Receptors
RARB	Cytokine_Receptors
RARG	Cytokine_Receptors

ROBO1	Cytokine_Receptors
ROBO2	Cytokine_Receptors
ROBO3	Cytokine_Receptors
RORA	Cytokine_Receptors
RORB	Cytokine_Receptors
RORC	Cytokine_Receptors
RXFP1	Cytokine_Receptors
RXFP2	Cytokine_Receptors
RXFP3	Cytokine_Receptors
RXRA	Cytokine_Receptors
RXRB	Cytokine_Receptors
RXRG	Cytokine_Receptors
S1PR1	Cytokine_Receptors
S1PR2	Cytokine_Receptors
SCTR	Cytokine_Receptors
SDC1	Cytokine_Receptors
SDC2	Cytokine_Receptors
SDC3	Cytokine_Receptors
SDC4	Cytokine_Receptors
SORT1	Cytokine_Receptors
SSTR1	Cytokine_Receptors
SSTR2	Cytokine_Receptors
SSTR5	Cytokine_Receptors
ST2	Cytokine_Receptors
TACR1	Cytokine_Receptors
TEK	Cytokine_Receptors
TGFBR1	Cytokine_Receptors
TGFBR2	Cytokine_Receptors
TGFBR3	Cytokine_Receptors
THRA	Cytokine_Receptors
THRB	Cytokine_Receptors
TIE1	Cytokine_Receptors
TNFRSF10A	Cytokine_Receptors
TNFRSF10B	Cytokine_Receptors
TNFRSF10C	Cytokine_Receptors
TNFRSF10D	Cytokine_Receptors
TNFRSF11A	Cytokine_Receptors
TNFRSF12A	Cytokine_Receptors
TNFRSF13B	Cytokine_Receptors
TNFRSF13C	Cytokine_Receptors
TNFRSF14	Cytokine_Receptors
TNFRSF17	Cytokine_Receptors
TNFRSF18	Cytokine_Receptors
TNFRSF19	Cytokine_Receptors

TNFRSF1A	Cytokine_Receptors
TNFRSF1B	Cytokine_Receptors
TNFRSF21	Cytokine_Receptors
TNFRSF25	Cytokine_Receptors
TNFRSF4	Cytokine_Receptors
TNFRSF6B	Cytokine_Receptors
TNFRSF8	Cytokine_Receptors
TNFRSF9	Cytokine_Receptors
TRHR	Cytokine_Receptors
TSHR	Cytokine_Receptors
TUBB3	Cytokine_Receptors
VDR	Cytokine_Receptors
VIPR1	Cytokine_Receptors
VIPR2	Cytokine_Receptors
XCR1	Cytokine_Receptors
IFNA10	Interferons
IFNA13	Interferons
IFNA14	Interferons
IFNA16	Interferons
IFNA17	Interferons
IFNA2	Interferons
IFNA21	Interferons
IFNA4	Interferons
IFNA5	Interferons
IFNA6	Interferons
IFNA7	Interferons
IFNA8	Interferons
IFNB1	Interferons
IFNE	Interferons
IFNG	Interferons
IFNK	Interferons
IFNW1	Interferons
IFNAR2	Interferon_Receptor
IFNGR1	Interferon_Receptor
IFNGR2	Interferon_Receptor
IL11	Interleukins
IL12A	Interleukins
IL12B	Interleukins
IL13	Interleukins
IL15	Interleukins
IL16	Interleukins
IL17A	Interleukins
IL17B	Interleukins
IL17C	Interleukins

IL17D	Interleukins
IL17F	Interleukins
IL18	Interleukins
IL19	Interleukins
IL1A	Interleukins
IL1B	Interleukins
IL1F10	Interleukins
IL36RN	Interleukins
IL36A	Interleukins
IL37	Interleukins
IL36B	Interleukins
IL36G	Interleukins
IL1RN	Interleukins
IL2	Interleukins
IL20	Interleukins
IL21	Interleukins
IL22	Interleukins
IL23A	Interleukins
IL24	Interleukins
IL25	Interleukins
IL26	Interleukins
IL27	Interleukins
IFNL2	Interleukins
IFNL3	Interleukins
IFNL1	Interleukins
IL3	Interleukins
IL31	Interleukins
IL32	Interleukins
IL33	Interleukins
IL34	Interleukins
IL4	Interleukins
IL5	Interleukins
IL6	Interleukins
IL6ST	Interleukins
IL7	Interleukins
CXCL8	Interleukins
IL9	Interleukins
TXLNA	Interleukins
IL10RA	Interleukins_Receptor
IL10RB	Interleukins_Receptor
IL11RA	Interleukins_Receptor
IL12RB1	Interleukins_Receptor
IL12RB2	Interleukins_Receptor
IL13RA1	Interleukins_Receptor

IL13RA2	Interleukins_Receptor
IL15RA	Interleukins_Receptor
IL2RB	Interleukins_Receptor
IL17RA	Interleukins_Receptor
IL17RB	Interleukins_Receptor
IL17RC	Interleukins_Receptor
IL17RD	Interleukins_Receptor
IL17RE	Interleukins_Receptor
IL18R1	Interleukins_Receptor
IL18RAP	Interleukins_Receptor
IL1R1	Interleukins_Receptor
IL1R2	Interleukins_Receptor
IL1RAP	Interleukins_Receptor
IL1RL1	Interleukins_Receptor
IL1RL2	Interleukins_Receptor
IL20RA	Interleukins_Receptor
IL20RB	Interleukins_Receptor
IL21R	Interleukins_Receptor
IL22RA1	Interleukins_Receptor
IL22RA2	Interleukins_Receptor
IL23R	Interleukins_Receptor
IL27RA	Interleukins_Receptor
IFNLR1	Interleukins_Receptor
IL2RA	Interleukins_Receptor
IL2RB	Interleukins_Receptor
IL2RG	Interleukins_Receptor
IL31RA	Interleukins_Receptor
IL3RA	Interleukins_Receptor
IL4R	Interleukins_Receptor
IL5RA	Interleukins_Receptor
IL6R	Interleukins_Receptor
IL7R	Interleukins_Receptor
CXCR1	Interleukins_Receptor
CXCR2	Interleukins_Receptor
IL9R	Interleukins_Receptor
ST2	Interleukins_Receptor
HLA-A	NaturalKiller_Cell_Cytotoxicity
HLA-B	NaturalKiller_Cell_Cytotoxicity
HLA-C	NaturalKiller_Cell_Cytotoxicity
HLA-E	NaturalKiller_Cell_Cytotoxicity
HLA-G	NaturalKiller_Cell_Cytotoxicity
KIR3DL1	NaturalKiller_Cell_Cytotoxicity
KIR3DL2	NaturalKiller_Cell_Cytotoxicity
KIR2DL1	NaturalKiller_Cell_Cytotoxicity

KIR2DL2	NaturalKiller_Cell_Cytotoxicity
KIR2DL3	NaturalKiller_Cell_Cytotoxicity
KIR2DL4	NaturalKiller_Cell_Cytotoxicity
KIR2DL5A	NaturalKiller_Cell_Cytotoxicity
KLRC1	NaturalKiller_Cell_Cytotoxicity
KLRC2	NaturalKiller_Cell_Cytotoxicity
KLRC3	NaturalKiller_Cell_Cytotoxicity
KLRD1	NaturalKiller_Cell_Cytotoxicity
PTPN6	NaturalKiller_Cell_Cytotoxicity
PTPN11	NaturalKiller_Cell_Cytotoxicity
ICAM1	NaturalKiller_Cell_Cytotoxicity
ICAM2	NaturalKiller_Cell_Cytotoxicity
ITGAL	NaturalKiller_Cell_Cytotoxicity
ITGB2	NaturalKiller_Cell_Cytotoxicity
PTK2B	NaturalKiller_Cell_Cytotoxicity
VAV3	NaturalKiller_Cell_Cytotoxicity
VAV1	NaturalKiller_Cell_Cytotoxicity
VAV2	NaturalKiller_Cell_Cytotoxicity
RAC1	NaturalKiller_Cell_Cytotoxicity
RAC2	NaturalKiller_Cell_Cytotoxicity
RAC3	NaturalKiller_Cell_Cytotoxicity
PAK1	NaturalKiller_Cell_Cytotoxicity
MAP2K1	NaturalKiller_Cell_Cytotoxicity
MAP2K2	NaturalKiller_Cell_Cytotoxicity
MAPK1	NaturalKiller_Cell_Cytotoxicity
MAPK3	NaturalKiller_Cell_Cytotoxicity
TNF	NaturalKiller_Cell_Cytotoxicity
CSF2	NaturalKiller_Cell_Cytotoxicity
IFNG	NaturalKiller_Cell_Cytotoxicity
KIR2DS1	NaturalKiller_Cell_Cytotoxicity
KIR2DS3	NaturalKiller_Cell_Cytotoxicity
KIR2DS4	NaturalKiller_Cell_Cytotoxicity
KIR2DS5	NaturalKiller_Cell_Cytotoxicity
NCR2	NaturalKiller_Cell_Cytotoxicity
TYROBP	NaturalKiller_Cell_Cytotoxicity
LCK	NaturalKiller_Cell_Cytotoxicity
FCGR3A	NaturalKiller_Cell_Cytotoxicity
FCGR3B	NaturalKiller_Cell_Cytotoxicity
NCR1	NaturalKiller_Cell_Cytotoxicity
NCR3	NaturalKiller_Cell_Cytotoxicity
FCER1G	NaturalKiller_Cell_Cytotoxicity
CD247	NaturalKiller_Cell_Cytotoxicity
ZAP70	NaturalKiller_Cell_Cytotoxicity
SYK	NaturalKiller_Cell_Cytotoxicity

LCP2	NaturalKiller_Cell_Cytotoxicity
LAT	NaturalKiller_Cell_Cytotoxicity
PLCG1	NaturalKiller_Cell_Cytotoxicity
PLCG2	NaturalKiller_Cell_Cytotoxicity
SH3BP2	NaturalKiller_Cell_Cytotoxicity
PIK3CA	NaturalKiller_Cell_Cytotoxicity
PIK3CB	NaturalKiller_Cell_Cytotoxicity
PIK3CD	NaturalKiller_Cell_Cytotoxicity
PIK3CG	NaturalKiller_Cell_Cytotoxicity
PIK3R5	NaturalKiller_Cell_Cytotoxicity
PIK3R1	NaturalKiller_Cell_Cytotoxicity
PIK3R2	NaturalKiller_Cell_Cytotoxicity
PIK3R3	NaturalKiller_Cell_Cytotoxicity
FYN	NaturalKiller_Cell_Cytotoxicity
SHC2	NaturalKiller_Cell_Cytotoxicity
SHC4	NaturalKiller_Cell_Cytotoxicity
SHC3	NaturalKiller_Cell_Cytotoxicity
SHC1	NaturalKiller_Cell_Cytotoxicity
GRB2	NaturalKiller_Cell_Cytotoxicity
SOS1	NaturalKiller_Cell_Cytotoxicity
SOS2	NaturalKiller_Cell_Cytotoxicity
HRAS	NaturalKiller_Cell_Cytotoxicity
KRAS	TCRsignalingPathway
NRAS	TCRsignalingPathway
FOS	TCRsignalingPathway
JUN	TCRsignalingPathway
CARD11	TCRsignalingPathway
BCL10	TCRsignalingPathway
MALT1	TCRsignalingPathway
CHUK	TCRsignalingPathway
IKBKB	TCRsignalingPathway
IKBKG	TCRsignalingPathway
NFKB1	TCRsignalingPathway
RELA	TCRsignalingPathway
NFKBIA	TCRsignalingPathway
NFKBIB	TCRsignalingPathway
NFKBIE	TCRsignalingPathway
CD28	TCRsignalingPathway
ICOS	TCRsignalingPathway
CD40LG	TCRsignalingPathway
PIK3R5	TCRsignalingPathway
PIK3R1	TCRsignalingPathway
PIK3R2	TCRsignalingPathway
PIK3R3	TCRsignalingPathway

PIK3CA	TCRsignalingPathway
PIK3CB	TCRsignalingPathway
PIK3CD	TCRsignalingPathway
PIK3CG	TCRsignalingPathway
AKT3	TCRsignalingPathway
AKT1	TCRsignalingPathway
AKT2	TCRsignalingPathway
MAP3K8	TCRsignalingPathway
MAP3K14	TCRsignalingPathway
PDCD1	TCRsignalingPathway
CTLA4	TCRsignalingPathway
PTPN6	TCRsignalingPathway
CBLC	TCRsignalingPathway
CBL	TCRsignalingPathway
CBLB	TCRsignalingPathway
IL2	TCRsignalingPathway
IL4	TCRsignalingPathway
IL5	TCRsignalingPathway
IL10	TCRsignalingPathway
IFNG	TCRsignalingPathway
CSF2	TCRsignalingPathway
TNF	TCRsignalingPathway
CDK4	TCRsignalingPathway
RASGRP1	TCRsignalingPathway
PDK1	TCRsignalingPathway
PLCG1	TCRsignalingPathway
PRKCQ	TCRsignalingPathway
TRAC	TCRsignalingPathway
TRAJ1	TCRsignalingPathway
TRAJ2	TCRsignalingPathway
TRAJ3	TCRsignalingPathway
TRAJ4	TCRsignalingPathway
TRAJ5	TCRsignalingPathway
TRAJ6	TCRsignalingPathway
TRAJ7	TCRsignalingPathway
TRAJ8	TCRsignalingPathway
TRAJ9	TCRsignalingPathway
TRAJ10	TCRsignalingPathway
TRAJ11	TCRsignalingPathway
TRAJ12	TCRsignalingPathway
TRAJ13	TCRsignalingPathway
TRAJ14	TCRsignalingPathway
TRAJ15	TCRsignalingPathway
TRAJ16	TCRsignalingPathway

TRAJ17	TCRsignalingPathway
TRAJ18	TCRsignalingPathway
TRAJ19	TCRsignalingPathway
TRAJ20	TCRsignalingPathway
TRAJ21	TCRsignalingPathway
TRAJ22	TCRsignalingPathway
TRAJ23	TCRsignalingPathway
TRAJ24	TCRsignalingPathway
TRAJ25	TCRsignalingPathway
TRAJ26	TCRsignalingPathway
TRAJ27	TCRsignalingPathway
TRAJ28	TCRsignalingPathway
TRAJ29	TCRsignalingPathway
TRAJ30	TCRsignalingPathway
TRAJ31	TCRsignalingPathway
TRAJ32	TCRsignalingPathway
TRAJ33	TCRsignalingPathway
TRAJ34	TCRsignalingPathway
TRAJ35	TCRsignalingPathway
TRAJ36	TCRsignalingPathway
TRAJ37	TCRsignalingPathway
TRAJ38	TCRsignalingPathway
TRAJ39	TCRsignalingPathway
TRAJ40	TCRsignalingPathway
TRAJ41	TCRsignalingPathway
TRAJ42	TCRsignalingPathway
TRAJ43	TCRsignalingPathway
TRAJ44	TCRsignalingPathway
TRAJ45	TCRsignalingPathway
TRAJ46	TCRsignalingPathway
TRAJ47	TCRsignalingPathway
TRAJ48	TCRsignalingPathway
TRAJ49	TCRsignalingPathway
TRAJ50	TCRsignalingPathway
TRAJ52	TCRsignalingPathway
TRAJ53	TCRsignalingPathway
TRAJ54	TCRsignalingPathway
TRAJ56	TCRsignalingPathway
TRAJ57	TCRsignalingPathway
TRAJ58	TCRsignalingPathway
TRAJ59	TCRsignalingPathway
TRAJ61	TCRsignalingPathway
TRAV1-1	TCRsignalingPathway
TRAV1-2	TCRsignalingPathway

TRAV2	TCRsignalingPathway
TRAV3	TCRsignalingPathway
TRAV4	TCRsignalingPathway
TRAV5	TCRsignalingPathway
TRAV7	TCRsignalingPathway
TRAV8-1	TCRsignalingPathway
TRAV8-2	TCRsignalingPathway
TRAV8-3	TCRsignalingPathway
TRAV8-4	TCRsignalingPathway
TRAV8-6	TCRsignalingPathway
TRAV8-7	TCRsignalingPathway
TRAV9-1	TCRsignalingPathway
TRAV9-2	TCRsignalingPathway
TRAV10	TCRsignalingPathway
TRAV12-1	TCRsignalingPathway
TRAV12-2	TCRsignalingPathway
TRAV12-3	TCRsignalingPathway
TRAV13-1	TCRsignalingPathway
TRAV13-2	TCRsignalingPathway
TRAV14DV4	TCRsignalingPathway
TRAV16	TCRsignalingPathway
TRAV17	TCRsignalingPathway
TRAV18	TCRsignalingPathway
TRAV19	TCRsignalingPathway
TRAV20	TCRsignalingPathway
TRAV21	TCRsignalingPathway
TRAV22	TCRsignalingPathway
TRAV23DV6	TCRsignalingPathway
TRAV24	TCRsignalingPathway
TRAV25	TCRsignalingPathway
TRAV26-1	TCRsignalingPathway
TRAV26-2	TCRsignalingPathway
TRAV27	TCRsignalingPathway
TRAV29DV5	TCRsignalingPathway
TRAV30	TCRsignalingPathway
TRAV34	TCRsignalingPathway
TRAV35	TCRsignalingPathway
TRAV36DV7	TCRsignalingPathway
TRAV38-1	TCRsignalingPathway
TRAV38-2DV8	TCRsignalingPathway
TRAV39	TCRsignalingPathway
TRAV40	TCRsignalingPathway
TRAV41	TCRsignalingPathway
TRBC1	TCRsignalingPathway

TRBC2	TCRsignalingPathway
TRBD1	TCRsignalingPathway
TRBD2	TCRsignalingPathway
TRBJ1-1	TCRsignalingPathway
TRBJ1-2	TCRsignalingPathway
TRBJ1-3	TCRsignalingPathway
TRBJ1-4	TCRsignalingPathway
TRBJ1-5	TCRsignalingPathway
TRBJ1-6	TCRsignalingPathway
TRBJ2-1	TCRsignalingPathway
TRBJ2-2	TCRsignalingPathway
TRBJ2-3	TCRsignalingPathway
TRBJ2-4	TCRsignalingPathway
TRBJ2-5	TCRsignalingPathway
TRBJ2-6	TCRsignalingPathway
TRBJ2-7	TCRsignalingPathway
TRBV2	TCRsignalingPathway
TRBV3-1	TCRsignalingPathway
TRBV4-1	TCRsignalingPathway
TRBV4-2	TCRsignalingPathway
TRBV4-3	TCRsignalingPathway
TRBV5-1	TCRsignalingPathway
TRBV5-4	TCRsignalingPathway
TRBV5-5	TCRsignalingPathway
TRBV5-6	TCRsignalingPathway
TRBV5-7	TCRsignalingPathway
TRBV5-8	TCRsignalingPathway
TRBV6-1	TCRsignalingPathway
TRBV6-2	TCRsignalingPathway
TRBV6-3	TCRsignalingPathway
TRBV6-4	TCRsignalingPathway
TRBV6-5	TCRsignalingPathway
TRBV6-6	TCRsignalingPathway
TRBV6-7	TCRsignalingPathway
TRBV6-8	TCRsignalingPathway
TRBV6-9	TCRsignalingPathway
TRBV7-2	TCRsignalingPathway
TRBV7-3	TCRsignalingPathway
TRBV7-4	TCRsignalingPathway
TRBV7-6	TCRsignalingPathway
TRBV7-7	TCRsignalingPathway
TRBV7-8	TCRsignalingPathway
TRBV7-9	TCRsignalingPathway
TRBV9	TCRsignalingPathway

TRBV10-1	TCRsignalingPathway
TRBV10-2	TCRsignalingPathway
TRBV10-3	TCRsignalingPathway
TRBV11-1	TCRsignalingPathway
TRBV11-2	TCRsignalingPathway
TRBV11-3	TCRsignalingPathway
TRBV12-3	TCRsignalingPathway
TRBV12-4	TCRsignalingPathway
TRBV12-5	TCRsignalingPathway
TRBV13	TCRsignalingPathway
TRBV14	TCRsignalingPathway
TRBV15	TCRsignalingPathway
TRBV16	TCRsignalingPathway
TRBV17	TCRsignalingPathway
TRBV18	TCRsignalingPathway
TRBV19	TCRsignalingPathway
TRBV20-1	TCRsignalingPathway
TRBV24-1	TCRsignalingPathway
TRBV25-1	TCRsignalingPathway
TRBV27	TCRsignalingPathway
TRBV28	TCRsignalingPathway
TRBV29-1	TCRsignalingPathway
TRBV30	TCRsignalingPathway
TRDC	TCRsignalingPathway
TRDD1	TCRsignalingPathway
TRDD2	TCRsignalingPathway
TRDD3	TCRsignalingPathway
TRDJ1	TCRsignalingPathway
TRDJ2	TCRsignalingPathway
TRDJ3	TCRsignalingPathway
TRDJ4	TCRsignalingPathway
TRDV1	TCRsignalingPathway
TRDV2	TCRsignalingPathway
TRDV3	TCRsignalingPathway
TRGV9	TCRsignalingPathway
TRGV8	TCRsignalingPathway
TRGV5	TCRsignalingPathway
TRGV4	TCRsignalingPathway
TRGV3	TCRsignalingPathway
TRGV2	TCRsignalingPathway
TRGJP2	TCRsignalingPathway
TRGJP1	TCRsignalingPathway
TRGJP	TCRsignalingPathway
TRGJ2	TCRsignalingPathway

TRGJ1	TCRsignalingPathway
TRGC2	TCRsignalingPathway
TRGC1	TCRsignalingPathway
TRAV6	TCRsignalingPathway
BMP1	TGFb_Family_Member
BMP10	TGFb_Family_Member
BMP15	TGFb_Family_Member
BMP2	TGFb_Family_Member
BMP3	TGFb_Family_Member
BMP4	TGFb_Family_Member
BMP5	TGFb_Family_Member
BMP6	TGFb_Family_Member
BMP7	TGFb_Family_Member
BMP8A	TGFb_Family_Member
BMP8B	TGFb_Family_Member
GDF1	TGFb_Family_Member
GDF10	TGFb_Family_Member
GDF11	TGFb_Family_Member
GDF15	TGFb_Family_Member
GDF2	TGFb_Family_Member
GDF3	TGFb_Family_Member
GDF5	TGFb_Family_Member
GDF6	TGFb_Family_Member
GDF7	TGFb_Family_Member
GDF9	TGFb_Family_Member
GDNF	TGFb_Family_Member
INHA	TGFb_Family_Member
INHBA	TGFb_Family_Member
INHBB	TGFb_Family_Member
INHBC	TGFb_Family_Member
INHBE	TGFb_Family_Member
LEFTY1	TGFb_Family_Member
LEFTY2	TGFb_Family_Member
NODAL	TGFb_Family_Member
TGFB1	TGFb_Family_Member
TGFB2	TGFb_Family_Member
TGFB3	TGFb_Family_Member
ACVR1B	TGFb_Family_Member_Receptor
ACVR1C	TGFb_Family_Member_Receptor
ACVR2A	TGFb_Family_Member_Receptor
ACVR2B	TGFb_Family_Member_Receptor
ACVRL1	TGFb_Family_Member_Receptor
AMHR2	TGFb_Family_Member_Receptor
BMPR1A	TGFb_Family_Member_Receptor

BMPR1B	TGFb_Family_Member_Receptor
BMPR2	TGFb_Family_Member_Receptor
TGFBR1	TGFb_Family_Member_Receptor
TGFBR2	TGFb_Family_Member_Receptor
TGFBR3	TGFb_Family_Member_Receptor
TNFRSF11B	TNF_Family_Members
TNFSF10	TNF_Family_Members
TNFSF11	TNF_Family_Members
TNFSF12	TNF_Family_Members
TNFSF13	TNF_Family_Members
TNFSF13B	TNF_Family_Members
TNFSF14	TNF_Family_Members
TNFSF15	TNF_Family_Members
TNFSF18	TNF_Family_Members
TNFSF4	TNF_Family_Members
TNFSF8	TNF_Family_Members
TNFSF9	TNF_Family_Members
TNFRSF10B	TNF_Family_Members_Receptors
TNFRSF10C	TNF_Family_Members_Receptors
TNFRSF10D	TNF_Family_Members_Receptors
TNFRSF11A	TNF_Family_Members_Receptors
TNFRSF12A	TNF_Family_Members_Receptors
TNFRSF13B	TNF_Family_Members_Receptors
TNFRSF13C	TNF_Family_Members_Receptors
TNFRSF14	TNF_Family_Members_Receptors
TNFRSF17	TNF_Family_Members_Receptors
TNFRSF18	TNF_Family_Members_Receptors
TNFRSF19	TNF_Family_Members_Receptors
TNFRSF1A	TNF_Family_Members_Receptors
TNFRSF1B	TNF_Family_Members_Receptors
TNFRSF21	TNF_Family_Members_Receptors
TNFRSF25	TNF_Family_Members_Receptors
TNFRSF4	TNF_Family_Members_Receptors
TNFRSF6B	TNF_Family_Members_Receptors
TNFRSF8	TNF_Family_Members_Receptors
TNFRSF9	TNF_Family_Members_Receptors
